# Maintenance therapy with a poly(ADP-ribose) polymerase inhibitor in patients with newly diagnosed advanced epithelial ovarian cancer: individual patient data and trial-level meta-analysis☆

**DOI:** 10.1016/j.esmoop.2022.100558

**Published:** 2022-08-22

**Authors:** S. Gulia, S. Kannan, J. Ghosh, S. Rath, A. Maheshwari, S. Gupta

**Affiliations:** 1 Department of Medical Oncology, Tata Memorial Centre,Â Mumbai, India; 2 Homi Bhabha National Institute, Mumbai, India; 3 Biostatistics, Tata Memorial Centre, Mumbai, India; 4 Gynecologic Oncology, Tata Memorial Centre, Mumbai, India

**Keywords:** PARP inhibitors, first-line, epithelial ovarian cancer, meta-analysis

## Abstract

**Background:**

We synthesize the efficacy and toxicity of poly(ADP-ribose) polymerase inhibitors (PARPis) in patients with newly diagnosed advanced ovarian cancer.

**Patients and methods:**

We manually extracted individual patient data (IPD) for progression-free survival (PFS) from published survival curves of randomized controlled trials (RCTs) that compared PARPi versus placebo as maintenance therapy in first-line treatment, for whole study populations and subgroups, based on BRCA1/BRCA2 mutation (germline and/or somatic) and homologous recombination deficiency (HRD) status, using WebPlotDigitizer software. The respective PFS curves for each study and combined population were reconstructed from extracted IPD. The primary outcome was PFS in combined whole population and subgroups.

**Results:**

In IPD analysis of combined population from three RCTs, with 2296 patients and 1287 events, PFS was significantly longer in PARPi versus placebo [median 20.4 (95% confidence interval (CI) 18.6-21.9) versus 14.9 (95% CI 13.9-16.5) months, respectively; hazard ratio (HR) 0.67, 95% CI 0.60-0.75; *P* < 0.001]. In IPD subgroup analyses from four eligible RCTs (2687 patients and 1485 events), median PFS was significantly longer in PARPi versus placebo arm, in the BRCA-mutated (45.7 versus 17.7 months, respectively; HR 0.38, 95% CI 0.32-0.46; *P* < 0.001), HRD-positive including BRCA-mutated (34.7 versus 17.9 months, respectively; HR 0.45, 95% CI 0.38-0.54; *P* < 0.001), and HRD positive excluding BRCA-mutated (22.3 versus 13.1 months, respectively; HR 0.47, 95% CI 0.34-0.65; *P* < 0.001) subgroups, but not in the HRD-negative (15.0 versus 11.3 months, respectively; HR 0.90, 95% CI 0.76-1.05; *P* = 0.75) subgroup. Results of trial-level meta-analysis were concordant with IPD analysis in whole population and subgroups.

**Conclusions:**

Among newly diagnosed ovarian cancer patients, PARPi maintenance therapy significantly improves PFS in those with germline and/or somatic BRCA mutation and/or HRD-positive tumor but not in those with HRD-negative tumor.

## Highlights

• Maintenance PARPi resulted in significant PFS improvement in total population, but benefit varied in subgroups.
• PARPi showed PFS gain in *BRCA*-mutated (45.7 versus 17.7 m) and HRD-positive subgroups.
• No significant PFS benefit was seen in the HRD-negative subgroup (15.0 versus 11.3 months; *P* = 0.75).
• PARPi should be a standard treatment in newly diagnosed ovarian cancer patients except those with HRD-negative tumors.

## Introduction

The standard treatment of newly diagnosed epithelial ovarian cancer is surgical cytoreduction followed by platinum–taxane chemotherapy.^1^ In patients with advanced stage disease, neoadjuvant chemotherapy followed by interval debulking surgery is often practiced, based on its noninferiority compared with primary debulking surgery.^2^, ^3^, ^4^ However, up to 80% of patients with advanced ovarian cancer experience disease relapse.^5^ Various strategies to improve outcomes in first-line setting, such as weekly administration of paclitaxel, intraperitoneal chemotherapy, and use of bevacizumab, have yielded limited successs.^6^, ^7^, ^8^, ^9^

Approximately 10%-20% of ovarian cancer patients have pathogenic or likely pathogenic germline variants (formerly called mutations) in *BRCA1* or *BRCA2* genes^10^^,^^11^ and ∼50% have somatic (tumor) defects in the homologous recombination repair pathway [homologous recombination deficiency (HRD)].^10^ These genomic findings have been associated with higher response to platinum chemotherapy and better outcomes.^12^

Poly(ADP-ribose) polymerase (PARP) is an enzyme involved in single-stranded DNA repair and its inhibition has been demonstrated to result in synthetic lethality in tumor cells that harbor germline or acquired DNA repair defects, including HRD.^13^ This therapeutic strategy has resulted in a class of drugs called PARP inhibitors (PARPis), which were initially approved as maintenance therapy in patients with recurrent ovarian cancer after response to platinum-based chemotherapy.^14^, ^15^, ^16^ Subsequently, PARPis have also been investigated in ovarian cancer in the first-line setting in several randomized controlled trials (RCTs)^17^, ^18^, ^19^, ^20^, ^21^, ^22^ and have been shown to improve progression-free survival (PFS), especially in patients with germline *BRCA1* or *BRCA2* mutations. These studies have included patients with or without germline *BRCA1* or *BRCA2* mutations and some have also performed testing on somatic tissue for *BRCA1* and *BRCA2* mutations and HRD. Therefore, the study populations comprise heterogenous subgroups with respect to genomic aberrations that could interact with the efficacy of PARPis.

Some previous meta-analyses have evaluated the efficacy of PARPi in the first-line setting but have only used trial-level data.^23^, ^24^, ^25^ Therefore, results of these analyses cannot be used to estimate absolute benefits of PARPi. We have used a recently reported methodology to extract individual patient time-to-event data from published survival curves, which enabled us to estimate relative and absolute survival benefits of PARPi in combined whole study population and important subgroups.^26^^,^^27^ We conducted, and report here, an extracted individual patient data (IPD) and trial-level meta-analysis to synthesize the evidence for efficacy and toxicity of maintenance PARPi in first-line treatment of ovarian cancer patients, including molecular defined subgroups of interest.

## Methods

We performed an extracted IPD and trial-level meta-analysis of PARPi maintenance in first-line treatment of advanced ovarian cancer by extracting and synthesizing data from relevant RCTs. The advantages of the extracted IPD analysis include ability to estimate absolute differences in survival proportions and better analysis of subgroup data, while disadvantages include requirement of expertise, time, and effort. We followed the Preferred Reporting Items for Systematic Reviews and Meta-analysis (PRISMA) guidelines.

### Study selection

To be eligible, the trial had to be randomized and compare PARPi with placebo as maintenance therapy after completion of first-line platinum-based treatment in patients with stage III or IV epithelial ovarian, primary peritoneal, or fallopian tube cancer. Single-arm prospective studies, retrospective analyses and trials that included patients with recurrent disease were excluded. The inclusion and exclusion criteria based on the Population, Intervention, Comparison and Outcomes (PICOS) model are shown in Supplementary Table S1, available at https://doi.org/10.1016/j.esmoop.2022.100558.

### Search strategy

Eligible trials were identified using a computerized search of the following databases from January 2012 to September 2020: PubMed, Embase, and The Cochrane Library. We also searched abstracts and virtual meeting presentations of the following major oncology conferences: American Society of Clinical Oncology Annual Meetings, 2012-2020; European Society of Medical Oncology/European Cancer Organization Meetings, 2012-2020; International Gynecologic Cancer Society Meetings, 2012-2020, and the Society of Gynecologic Oncology Meetings, 2012-2020. The references of articles finally included in the analysis were reviewed and hand searched, if necessary. The search strategy was as follows: “(‘ovarian cancer’ OR ‘ovarian neoplasm’) AND (‘PARP inhibitor’ OR ‘PARPi’ OR ‘olaparib’ OR ‘niraparib’ OR ‘rucaparib’ OR ‘veliparib’ OR ‘Poly (ADP-Ribose) Polymerase Inhibitors’ OR ‘placebo’) AND (‘maintenance therapy’) AND (‘randomised’ OR ‘randomised’) AND (‘trial’)”. Two investigators (SeG and SK) independently reviewed the titles, abstracts and full texts to choose potentially relevant studies. Any disagreements were resolved by a discussion between them and the corresponding author (SG).

### Data extraction

Data were independently extracted by the joint first authors (SeG and SK) and disagreements, if any, were resolved by discussion between them and the corresponding author (SG). The following information was extracted from each selected trial: authors, publication year, number of patients in experimental (PARPi) and control (placebo) arms, number of patients by germline *BRCA1* and *BRCA2* mutation status, number of patients by somatic *BRCA1* and *BRCA2* mutation status, number of patients by combination of HRD and *BRCA1* or *BRCA2* mutation status (HRD positive including germline and/or somatic *BRCA* mutation positive, HRD positive excluding germline and/or somatic *BRCA* mutation positive, HRD negative), PFS hazard ratio (HR) and its 95% confidence interval (CI) in the whole study population and all reported subgroups, toxicities by study arm, quality of life indices by study arm, time to first subsequent therapy by study arm, and the second PFS (PFS2) by study arm.

### Individual patient data extraction

The WebPlotDigitizer software was used to extract data from published PFS Kaplan–Meier curves.^28^ These data were extracted manually for each trial through an iteration process until the extracted number of PFS events matched closely with the published ones at each time point. Data extraction quality was evaluated based on estimated and published PFS durations by study arm, hazard ratios with their 95% confidence intervals, year-wise event numbers by study arm, and duration of follow-up in each trial. Using these individual patient-level extracted data and published numbers at risk, we reconstructed PFS curves for each study using the STATA command ipdfc, published by Wei and Royston.^26^ The detailed methodology of data extraction is described in Supplementary Appendix, Section I, available at https://doi.org/10.1016/j.esmoop.2022.100558. The data extraction and synthesis process is complex and requires an expert statistician to achieve a high degree of accuracy.

### Extracted individual patient data meta-analysis in combined whole study populations

Extracted IPD for whole study populations from three eligible studies (PRIMA, PAOLA-1, and VELIA) were combined and PFS Kaplan–Meier curves were generated by study arm (PARPi versus placebo). The SOLO1 study included only patients with germline *BRCA1* or *BRCA2* mutation, therefore data from this study was not combined with whole study populations of the other three studies, which included patients with and without *BRCA* mutations. Data from the SOLO1 trial were combined with the *BRCA*-mutated subgroups of other three studies. The forest plot for PFS for the combined whole population was constructed using the extracted data of these three studies. One arm of the VELIA study used PARPi only during the period of first-line chemotherapy (*n* = 383), therefore data from this arm were excluded from the analysis.

We also estimated the number of events and proportion (with 95% CI) of patients surviving progression free at each time point (1, 2, and 3 years) from the combined IPD PFS curves.

### Extracted individual patient data analysis in subgroups

The included studies have variably reported results by germline and/or somatic *BRCA1* or *BRCA2* mutation status. Therefore, for this analysis, we defined *BRCA* mutation-positive status as the presence of pathogenic or likely pathogenic variants in *BRCA1* or *BRCA2* genes in germline and/or tumor tissue. We extracted IPD from published or presented^22^ PFS curves for the following patient subgroups defined by *BRCA* mutation and HRD status: with presence of *BRCA* mutation (four trials), with HRD-positive tumors including those with presence of *BRCA* mutation (three trials), with HRD-positive tumors excluding those with presence of *BRCA* mutation (two trials), and those with HRD-negative tumors (three trials). IPD by study arm were combined for these subgroups, with generation of PFS Kaplan–Meier curves.

### Trial-level analysis

Trial-level meta-analysis was performed for PFS in whole study populations, above described molecular subgroups defined by *BRCA* and HRD status, and some additional subgroups as follows: age (≤65, >65 years), stage (III, IV), response to treatment (complete response, partial response), and residual disease after surgery [nil residual (R0), macroscopic residual (R+)].

For each study, using the published PFS hazard ratios, we obtained O-E and V statistics as described by Tierney et al.^29^ HR estimates were pooled using a random-effects model due to heterogeneity among the studies. We generated forest plots for the effect of PARPi versus placebo in the whole population and subgroups.

### Statistical analysis

The study endpoint was comparison of PFS between PARPi and placebo in combined whole study populations (data from three trials) and in various molecular and clinical subgroups (data from three trials). The results were considered statistically significant if the upper or lower boundary of the 95% CI of the estimated HR of PARPi versus placebo did not cross unity, at a two-sided type I error of 0.05. The definition of PFS was consistent across the included trials (Supplementary Table S2, available at https://doi.org/10.1016/j.esmoop.2022.100558).

Summary estimates were reported as relative risk (RR) for binary outcomes and HR for time-to-event outcomes. All statistical analyses were performed using Review Manager 5.3 (Cochrane Collaboration, Copenhagen, Denmark) and STATA, version 14.0 (StataCorp, College Station, TX, USA).

The methodological quality of each eligible RCT was assessed using the Cochrane Collaboration Risk of Bias Tool under five domains: selection bias, performance bias, detection bias, attrition bias, and reporting bias.^30^ We also graded the quality of generated evidence based on the following parameters: risk of bias, imprecision, inconsistency, indirectness, and publication bias.^31^

### Patient and public involvement

No patients or members of the public were involved in any aspect of this meta-analysis, including setting the research question.

## Results

### Literature search and characteristics of included RCT and study population

The detailed criteria for inclusion or exclusion of data in the meta-analysis are presented in Supplementary Figure S1, available at https://doi.org/10.1016/j.esmoop.2022.100558. The initial search yielded 430 articles, of which four RCTs comparing PARPi and placebo as maintenance therapy after completion of first-line treatment were included in the final analysis. Among these studies, two tested olaparib (monotherapy in SOLO1 and with bevacizumab in PAOLA-1), one tested niraparib, and one tested veliparib as maintenance therapy in newly diagnosed advanced ovarian cancer patients. Table 1 lists the important characteristics of RCTs included in the meta-analysis. There were a total of 2687 patients in the included studies of whom 1666 (62%) and 1021 (38%) were allocated to the PARPi and the placebo arms, respectively. Supplementary Table S3, available at https://doi.org/10.1016/j.esmoop.2022.100558 describes the clinical, treatment, and genetic characteristics of the combined whole population included in the meta-analysis. The PARPi and placebo groups were balanced with respect to stage, receipt of neoadjuvant chemotherapy, tumor *BRCA* mutation, and HRD status, but there was a higher proportion of patients with germline *BRCA* mutation in the PARPi group (26%) compared with placebo (19%).

**Table 1:** Description of randomized trials included in the meta-analysis

Trial	*N* (experimental arm)	*N* (control arm)	Primary endpoint	Secondary endpoint	HR for PFS (95% CI) Whole population	HR for PFS (95% CI) germline BRCA mutated		HR for PFS (95% CI) tumor BRCA mutated		HR for PFS (95% CI) BRCA nonmutated			HR for PFS (95% CI) HRD positive, including BRCA mutated		HR for PFS (95% CI) HRD positive, excluding BRCA mutated			HR for PFS (95% CI) HRD negative		HR for PFS (95% CI) HRD unknown				Median follow-up
						E/N (PARPi)	E/N (placebo)	E/N (PARPi)	E/N (placebo)	E/N (PARPi)		E/N (placebo)	E/N (PARPi	E/N (placebo)	E/N (PARPi	E/N (placebo)		E/N (PARPi	E/N (placebo)	E/N (PARPi	E/N (placebo)			
Moore K^17^ (SOLO1)	260 Olaparib	131 Placebo	PFS	OS, PFS2 Time until first and second subsequent therapies Quality of life	—	118/260	100/131	—		—			—		—			—		—				60 months
						0.33 (0.024-0.43)																		
Martin AG^19^ (PRIMA)	487 Niraparib	246 Placebo	PFS	OS, PFS2 Time until subsequent therapy Quality of life	0.62 (0.50-0.76)	—		—		—			49/152	40/71	32/95		33/55	111/169	56/80	40/71			26/40	13.8 months
													0.40 (0.27-0.62)		0.50 (0.31-0.83)			0.68 (0.49-0.94)		0.85 (0.51-1.43)				
Coleman RL^20^ (VELIA)	382 Veliparib throughout group 383 Veliparib combination only group	375 Placebo	PFS in veliparib throughout group versus control	OS, PFS, and OS in the veliparib combination only group versus control Disease-related symptom score	0.68 (0.56-0.83)	27/80	36/63	7/28	15/29	142/245	171/254		87/214	124/207	—			80/125	89/124	—				28 months
						0.50 (0.30-0.82)		0.35 (0.14-0.87)		0.80 (0.64-1.00)			0.58 (0.44-0.76)					0.81 (0.60-1.09)						
Coquard IR^21^ (PAOLA-1)	537 Olaparib + bevacizumab	269 Placebo + bevacizumab	PFS	OS, PFS2 Time until subsequent therapy Quality of life	0.59 (0.49-0.72)	—		41/157	49/80	239/380	145/189		87/255	92/132	43/97		40/55	145/192	66/85	48/90		36//52		22.9 months
								0.31 (0.20-0.47)		0.71 (0.58-0.88)			0.33 (0.25-0.45)		0.43 (0.28-0.66)			0.92 (0.72-1.17		0.71 (0.46-1.10)				

Germline BRCA mutated: includes patients with germline BRCA1 and or BRCA2 mutation positive; Tumor BRCA mutated: includes patients with tumor BRCA 1 and/or BRCA 2 mutation positive; BRCA nonmutated: includes patients with germline and tumor BRCA1/2 nonmutated cases. Homologous repair deficiency was defined as either HRD score of ≥42 (in the PRIMA and POALA1 trials) or HRD score of ≥33 (in the VELIA trial) and/or deleterious BRCA1/2 mutation (germline or tumor). HRD negative was defined as HRD score <33 (in the VELIA trial) and <42 (in the PRIMA and POALA1 trials). HRD unknown was defined as inconclusive, missing, or failed test.

CI, confidence interval; E, number of events in the respective subgroups and treatment arm; HR, hazard ratio; HRD, homologous repair deficiency; *N*, number of patients in the respective subgroups and treatment arm; *N*, number of patients; OS, overall survival; PARPi, poly(ADP-ribose) polymerase inhibitor; PFS, progression-free survival; PFS2, second progression-free survival.

### Risk of bias

Assessment of risk of bias in included studies is presented in Supplementary Figure S2, available at https://doi.org/10.1016/j.esmoop.2022.100558 and was low for all included studies. The quality of evidence was graded high for PFS (Supplementary Table S4, available at https://doi.org/10.1016/j.esmoop.2022.100558).

### Individual patient data extraction

The extracted and reported PFS events, hazard ratios, and median PFS in each of the four included RCT are shown in Supplementary Table S5, available at https://doi.org/10.1016/j.esmoop.2022.100558. The extracted and reported events were nearly identical to each other in all studies, indicating high accuracy of the IPD extraction methodology. The trial-wise PFS HR from extracted data did not match precisely with reported HR because our calculation was unadjusted while those reported in the four studies (SOLO1, PAOLA-1, PRIMA, and VELIA) were adjusted for various covariates.

### Extracted individual patient data progression-free survival analysis

**Figure 1:**
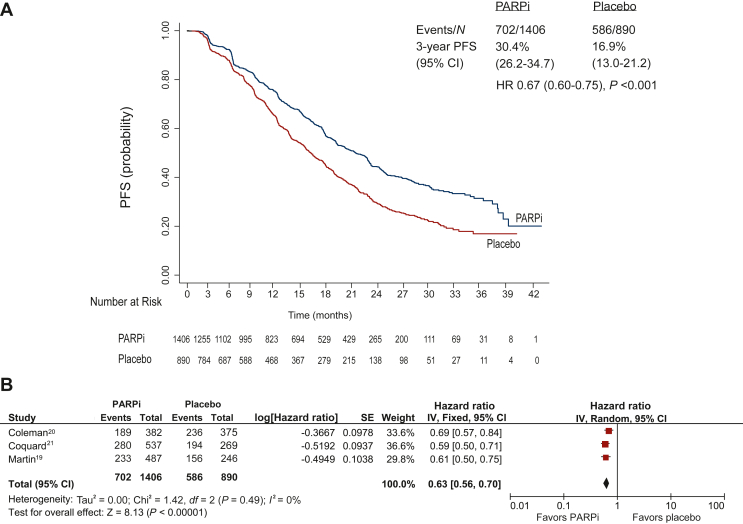
**Progression-free survival comparing PARPi versus placebo: Combined whole population individual patient data from three RCTs (excluding trial by Moore et al.**^17^**).** (A) Progression-free survival curve. (B) Forest plot. CI, confidence interval; HR, hazard ratio; PARPi, poly(ADP-ribose) polymerase inhibitor; PFS, progression-free survival; RCT, randomized controlled trial.

The individual patient analysis of PFS in the combined study population was performed in data extracted from three studies (PRIMA, PAOLA-1, and VELIA) with 1406 patients and 702 events in the PARPi group and 890 patients and 586 events in the placebo group. The median PFS was significantly longer in PARPi compared with placebo group [20.4 (95% CI 18.6-21.9) months versus 14.9 (95% CI 13.9-16.5) months; HR 0.67, 95% CI 0.60-0.75; *P* < 0.001], as shown in Figure 1A and B. The HR derived from Kaplan–Meier PFS curves did not match precisely with that derived from the forest plot, because clustering of patients within different studies is ignored in STATA while weightage is given to studies according to their sample size in Review Manager. The proportion of patients who remained progression free was significantly higher in the PARPi compared with the placebo group at 1 year [70.23% (95% CI 67.63% to 72.66%) versus 59.52% (95% CI 56.08% to 62.78%); *P* < 0.001], 2 years [41.91% (95% CI 38.82% to 44.97%) versus 27.94% (95% CI 24.64% to 31.33%); *P* < 0.001], and 3 years [30.43% (95% CI 26.20% to 34.77%) versus 16.90% (95% CI 13.06% to 21.18%); *P* < 0.001] (Supplementary Table S6, available at https://doi.org/10.1016/j.esmoop.2022.100558).

### Extracted individual patient data progression-free survival analysis in subgroups

**Figure 2:**
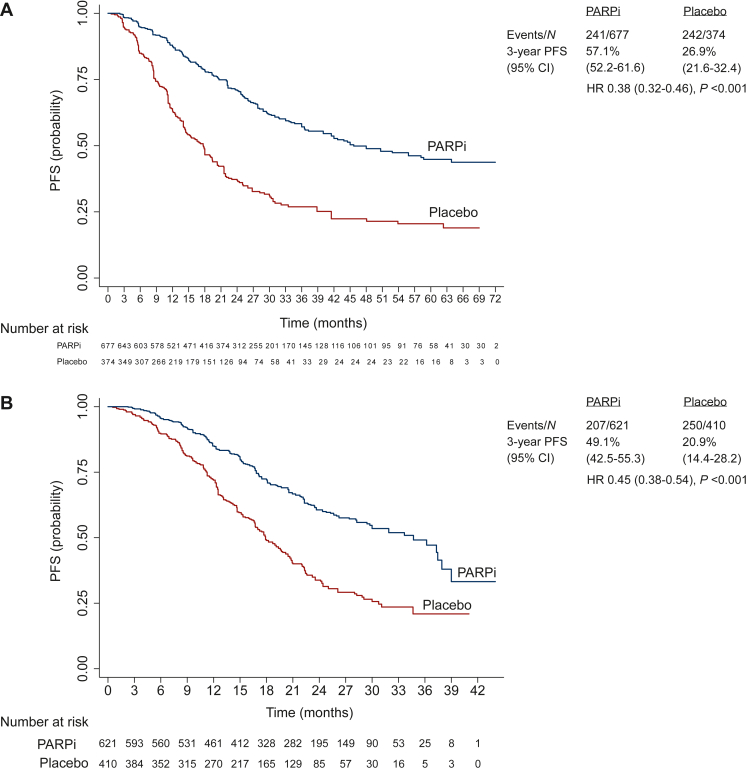

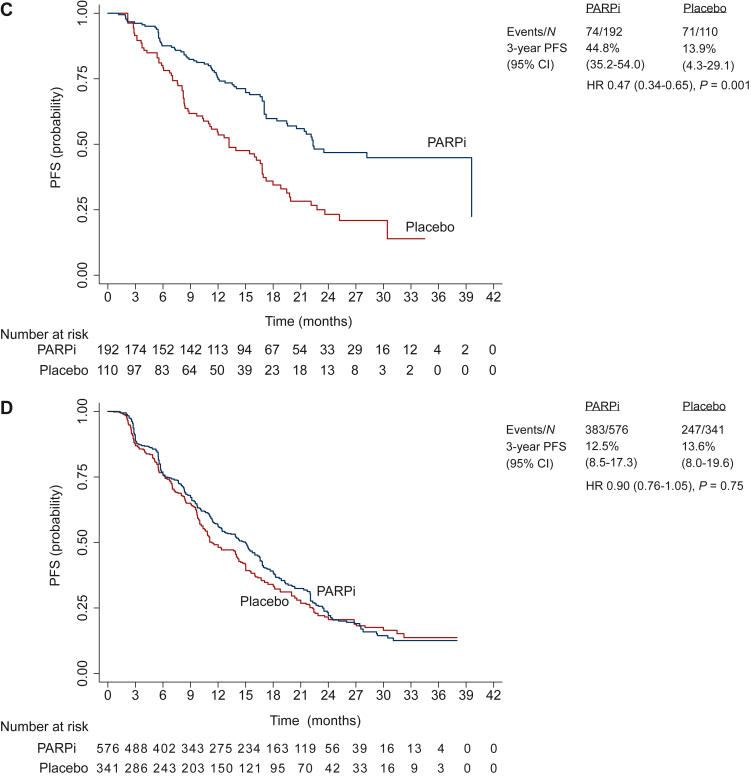
**Progression-free survival curves comparing PARPi versus placebo in subgroups: combined individual patient data from four RCTs.** (A) Germline and/or tumor *BRCA* mutated. (B) HRD positive including *BRCA* mutated. (C) HRD positive excluding *BRCA* mutated. (D) HRD negative.CI, confidence interval; HR, hazard ratio; HRD, homologous recombination deficiency; PARPi, poly(ADP-ribose) polymerase inhibitor; PFS, progression-free survival; RCT, randomized controlled trial.

**Figure 3:**
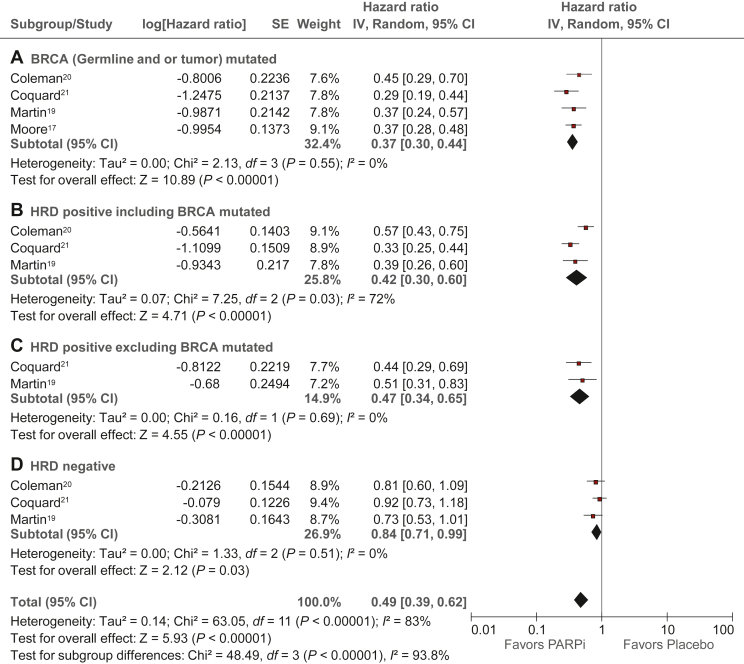
**Progression-free survival comparing PARPi versus placebo in subgroups: forest plots of individual patient data from four RCTs.** (A) Germline and/or tumor *BRCA* mutated. (B) HRD positive including *BRCA* mutated. (C) HRD positive excluding *BRCA* mutated. (D) HRD negative.CI, confidence interval; HRD, homologous recombination deficiency; M–H, Mantel–Haenszel; PARPi, poly(ADP-ribose) polymerase inhibitor; RCT, randomized controlled trial.

The individual patient analysis of PFS in relevant subgroups was performed in data extracted from four studies (PRIMA, PAOLA-1, VELIA, and SOLO-1) with 1505 patients and 732 events in the PARPi group and 929 patients and 624 events in the placebo group. The median PFS was significantly longer in PARPi versus placebo groups, among patients with germline and/or tumor *BRCA* mutation [45.7 (95% CI 40.0-63.8) months versus 17.7 (95% CI 14.4-19.4) months; HR 0.38, 95% CI 0.32-0.46; *P* < 0.001; *I*^2^ = 0%; Figures 2A and 3A], HRD-positive status including *BRCA* mutation [34.7 (95% CI 29.7-37.9) months versus 17.9 (95% CI 16.7-19.7) months; HR 0.45, 95% CI 0.38-0.54; *P* < 0.001; *I*^2^ = 72%; Figures 2B and 3B], and HRD-positive status excluding *BRCA* mutation [22.3 (95% CI 19.4-not-estimable) months versus 13.1 (95% CI 10.3-16.8) months; HR 0.47, 95% CI 0.34-0.65; *P* = 0.001; *I*^2^ = 0%; Figures 2C and 3C]. However, the median PFS was not significantly different in PARPi versus placebo groups among patients with HRD-negative status [15.0 (95% CI 12.4-16.0) months versus 11.3 (95% CI 10.3-14.0) months; HR 0.90, 95% CI 0.76-1.05; *P* = 0.75; *I*^2^ = 0%; Figures 2D and 3D].

### Trial-level progression-free survival analysis

The trial-level analysis of PFS in the combined study population (Supplementary Figure S3, available at https://doi.org/10.1016/j.esmoop.2022.100558) was performed from published data from three studies (PRIMA, PAOLA-1, and VELIA) with 1406 patients and 703 events in the PARPi group and 890 patients and 586 events in the placebo group. Patients in the PARPi group had significantly longer PFS compared with patients in the placebo group (HR 0.63, 95% CI 0.56 to 0.71).

### Trial-level progression-free survival analysis in subgroups

The trial-level analysis of PFS in relevant subgroups (Supplementary Figure S4, available at https://doi.org/10.1016/j.esmoop.2022.100558) was performed from published data from four studies (PRIMA, PAOLA-1, VELIA, and SOLO-1) with 1505 patients and 733 events in the PARPi group and 929 patients and 624 events in the placebo group. The median PFS was significantly longer in PARPi versus placebo groups, among patients with germline and/or tumor *BRCA* mutation (HR 0.36, 95% CI 0.30-0.43; *P* < 0.01; *I*^2^ = 0%), HRD-positive status including *BRCA* mutation (HR 0.42, 95% CI 0.30-0.60; *P* < 0.01; *I*^2^ = 71%), HRD-positive status excluding *BRCA* mutation (HR 0.46, 95% CI 0.33-0.63; *P* < 0.01; *I*^2^ = 0%), and HRD-negative tumor (HR 0.82, 95% CI 0.69 to 0.97; *P* = 0.02; *I*^2^ = 6%). Of note, significant heterogeneity (*I*^2^ = 71%, *P* = 0.03) was seen in the HRD-positive subgroup that included *BRCA* mutation. This could be due to different cut-off scores for HRD in the included trials (42 was the cut-off in PRIMA and PAOLA-1, and 33 in VELIA). There was no significant heterogeneity in the HRD-positive subgroup after excluding *BRCA* mutation because this analysis did not include the VELIA study.

The median PFS was significantly longer in PARPi versus placebo groups among clinically relevant subgroups of patients (Supplementary Figure S5, available at https://doi.org/10.1016/j.esmoop.2022.100558), including age [<65 years (HR 0.62, 95% CI 0.54-0.72) and ≥65 years (HR 0.61, 95% CI 0.48-0.77)], performance status [Eastern Cooperative Oncology Group performance status (ECOG PS) 0 (HR 0.65, 95% CI 0.56-0.74) and ECOG PS 1 (HR 0.61, 95% CI 0.51-0.74)], stage [III (HR 0.62, 95% CI 0.54-0.71) and IV (HR 0.66, 95% CI 0.48-0.92)], response to treatment [complete response (HR 0.54, 95% CI 0.40-0.72) and partial response (HR 0.72, 95% CI 0.51-1.03)], and residual disease after surgery [nil residual (HR 0.57, 95% CI 0.46-0.71) and macroscopic residual (HR 0.68, 95% CI 0.54-0.84)]. None of these subgroup analyses showed significant heterogeneity.

### Trial-level analysis of time-to-first-subsequent therapy and second progression-free survival

The trial-level analysis of time-to-first-subsequent therapy in combined study population (Supplementary Figure S6, available at https://doi.org/10.1016/j.esmoop.2022.100558) was performed in published data from two studies (PRIMA and PAOLA-1) and was significantly longer in the PARPi group compared with the placebo group (HR 0.61, 95% CI 0.53-0.71). The trial-level analysis of PFS2 in combined study population (Supplementary Figure 6A, available at https://doi.org/10.1016/j.esmoop.2022.100558) was performed in published data from two studies (PRIMA and PAOLA-1) and was borderline significantly longer in the PARPi group compared with the placebo group (HR 0.84, 95% CI 0.70-1.02), but these data are still immature (Supplementary Figure 6A, available at https://doi.org/10.1016/j.esmoop.2022.100558).

### Trial-level analysis of adverse events

**Figure 4:**
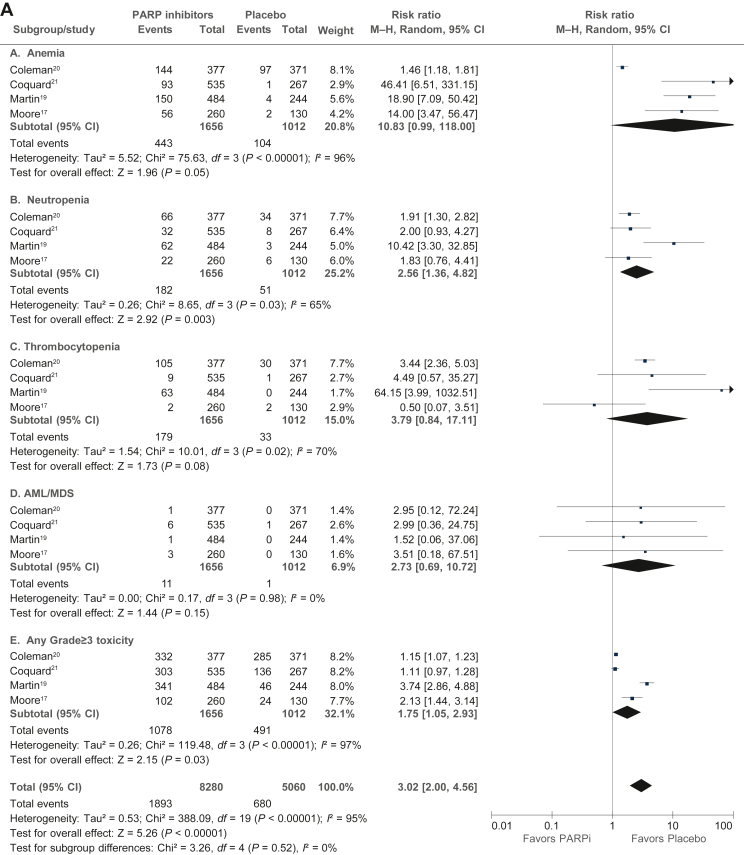

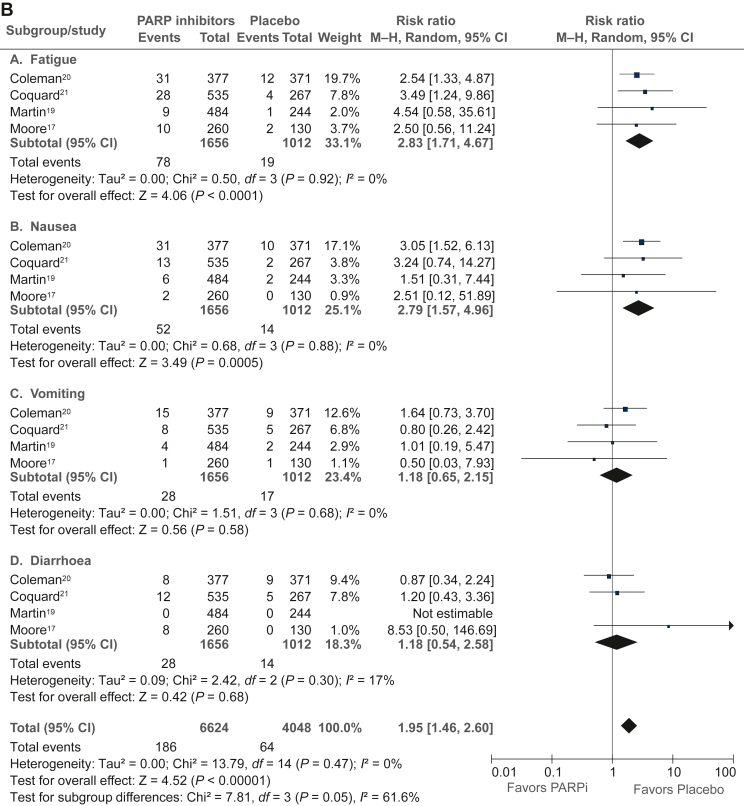

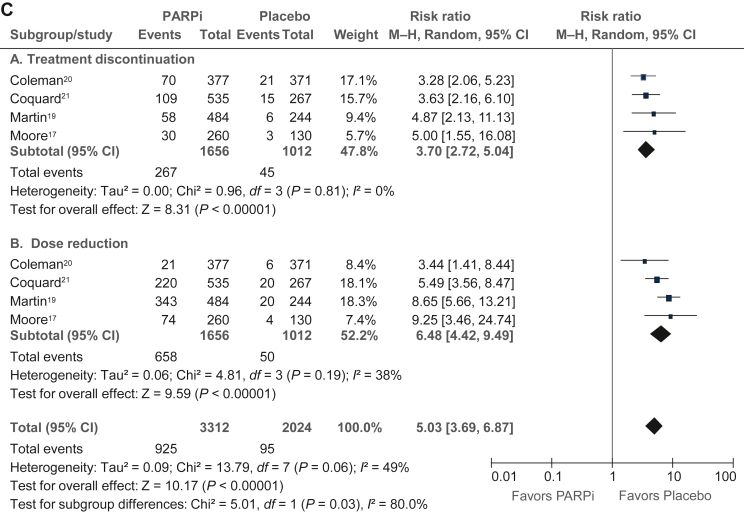
**Analysis of toxicities (≥grade III) in PARP inhibitor versus placebo based on published estimates.** (A) Hematological toxicities. (B) Nonhematological toxicities. (C) Dose reduction and treatment discontinuation. AML, acute myeloid leukemia; CI, confidence interval; MDS, myelodysplastic syndrome; M–H, Mantel–Haenszel; PARPi, poly(ADP-ribose) polymerase inhibitor.

The trial-level analysis of adverse events (Figure 4A-C) was performed in published data from four studies (PRIMA, PAOLA-1, VELIA, and SOLO-1) with 1656 patients in the PARPi group and 1012 patients in the placebo group. In PARPi versus placebo groups, there was a significantly higher risk of grade ≥3 any adverse event (RR 3.05, 95% CI 1.99-4.68), grade ≥3 nausea (RR 2.90, 95% CI 1.60-5.25), grade ≥3 fatigue (RR 2.97, 95% CI 1.76-4.99), grade ≥3 anemia (RR 13.57, 95% CI 1.57-121.11), grade ≥3 neutropenia (RR 2.78, 95% CI 1.44-5.39), grade ≥3 thrombocytopenia (RR 4.28, 95% CI 0.92-19.98), dose reduction (RR 6.48, 95% CI 4.42-9.49), treatment discontinuation (RR 3.70, 95% CI 2.72-5.04), and acute myeloid leukemia/myelodysplastic syndrome (RR 2.73, 95% CI 0.69-10.72). However, there was no difference between the PARPi and placebo groups in health-related quality-of-life evaluation in any of the four included studies (Supplementary Table S7, available at https://doi.org/10.1016/j.esmoop.2022.100558).

## Discussion

To our knowledge, this is the first and only meta-analysis to synthesize patient-level data from all relevant randomized studies of PARPis in the first-line treatment of patients with newly diagnosed advanced stage epithelial ovarian cancer. The result of extracted IPD meta-analysis of data from three studies, which included patients regardless of *BRCA* mutation status and HRD status, suggests that use of a PARPi as maintenance treatment after chemotherapy resulted in significantly longer PFS compared with placebo, with a 33% reduction in the risk of progression or death. Importantly, extracted IPD analysis in subgroups of patients defined by germline and/or tumor *BRCA* mutation status and HRD status, from four randomized studies, suggests that the benefit of PARPi is variable in these biologically defined subgroups. There was substantial relative and absolute PFS benefit of PARP inhibition in patients with germline and/or tumor *BRCA* mutation (62% reduction in risk, absolute gain 28.0 months), those whose tumors had HRD including *BRCA* mutation (55% reduction in risk, absolute gain 16.8 months), and in those with tumor HRD without *BRCA* mutation (53% reduction in risk, absolute gain 9.2 months), but there was no significant benefit in patients without HRD. The results of trial-level analyses were largely concordant with IPD analysis.

Our IPD analysis using accurately extracted IPD allows estimation of absolute benefit of PARPi as first-line maintenance strategy. This information could be particularly useful in counseling patients to participate in clinical decision making. The results of our analysis suggest that the magnitude of benefit of PARPis possibly lies along a gradient, with the presence of germline and/or tumor *BRCA* mutation associated with maximum benefit, followed by the presence of tumor HRD without *BRCA* mutation. There was a lack of statistically significant and clinically meaningful benefit of PARP inhibition in patients without tumor HRD, while other patients seemed to derive substantial benefit. This suggests that, were it to be more widely available and accessible, tumor HRD testing could be the first test to aid PARP inhibition-related therapeutic decision making in the first-line setting in ovarian cancer. The meta-analysis result in an HRD-negative population should be considered in the context of heterogeneity in the experimental arms of the three studies wherein this biomarker was used. Among patients with HRD-negative tumors, the 95% CI of PFS HR crossed unity in VELIA (PARPi started with chemotherapy) and PAOLA-1 (PARPi given with bevacizumab) but not in PRIMA (PARPi used as single-agent maintenance). Moreover, there was a variable cut-off value for defining the HRD cohort by the companion diagnostic test (myChoice HRD CDx assay),^32^^,^^33^ which was ≥42 in PRIMA and PAOLA-1 trials but ≥33 in the VELIA trial. This means that any meta-analysis which combines the data from these trials will have some genomic heterogeneity in the HRD-positive as well as HRD-negative cohorts. A patient-level reclassification of patients from all three trials by a uniform HRD cut-off value and subsequent combined analysis might further clarify the benefit of PARP inhibition in a more HRD homogenous population. It is also worth noting that 18% of patients in PAOLA-1 and 9% in PRIMA had inconclusive results of HRD testing. With all these caveats, it is clear from the results of this meta-analysis that patients with wild-type BRCA and HRD-negative tumors do not derive benefit from first-line PARPi maintenance treatment and that this treatment should not be a standard option in this subgroup.

An interesting observation from our analysis of combined subgroup populations is the gradient of median PFS in the control (placebo) arm in four *BRCA-* and HRD-defined subgroups (germline/tumor *BRCA* mutated 17.7 months, HRD positive including *BRCA* mutated 17.9 months, HRD positive excluding *BRCA* mutated 13.1 months, HRD negative 11.3 months). This suggests that the presence of HRD in the tumor (with or without germline/tumor *BRCA* mutation) is an inherent prognostic factor even with standard (no PARPi) treatment and that this (or similar) genetic/genomic characterization should possibly be incorporated as a stratification factor in most ovarian cancer clinical trials in the future.

The results of our trial-level meta-analyses in clinically relevant subgroups defined by age, stage (III or IV), level of response to chemotherapy (complete or partial), and amount of residual disease after surgery suggest that the benefit of PARP inhibition is maintained in all subgroups and these factors should not be used to choose patients for this therapeutic strategy.

As expected, collated trial-level analysis of combined data from four studies showed a significantly higher risk of several hematological and nonhematological toxicities in the PARPi group compared with placebo, which have to be considered in clinical decisions. There was a substantially increased risk of anemia and increased risk of fatigue, which are important considerations in a maintenance treatment strategy that is delivered over a long period. The risk of acute myeloid leukemia and myelodysplastic syndrome was higher with PARPi in our analysis, in concordance with a previous meta-analysis (odds ratio 2.63, 95% CI 1.13-6.14; *P* = 0.026),^34^ but low in absolute terms. Although we were unable to combine and meta-analyze the quality-of-life data from the included studies, none of them individually showed a detriment in QOL with PARPi.

The strength of our analysis is accurate extraction of IPD from whole study populations and relevant subgroups in each trial (Supplementary Figures S8-S24, available at https://doi.org/10.1016/j.esmoop.2022.100558) using a recently described methodology.^26^, ^27^ This enables estimation of absolute benefits of PARP inhibition in various subgroups of patients. The evaluation of germline and/or somatic *BRCA* mutations was relatively uniform in included studies, which allowed us to interrogate the efficacy of PARPis in biologically homogenous patient subgroups. We included only first-line trials of PARPis, unlike a previous meta-analysis,^25^ ensuring better homogeneity of patient population. By performing trial-level meta-analysis in addition to IPD analysis, we have attempted to reduce uncertainties around either result.

An ongoing question of clinical relevance is the sequencing of PARP inhibition in ovarian cancer, given the lack of mature overall survival data from first-line maintenance studies. The substantial magnitude of PFS benefit with PARPi in the first-line setting in BRCA-mutated and HRD-positive cohorts, lack of opportunity to use PARPis in a proportion of patients with recurrent disease,^5^ and the need for platinum sensitivity when using PARPis in the recurrent/relapsed setting suggest that first-line use may be the optimal strategy.^35^

There are some limitations of our analysis, mainly related to differing design characteristics of included studies with respect to patient eligibility, treatment characteristics, and statistical considerations. One study (PRIMA) excluded patients with stage III disease and nil residual disease after surgery, while the other three studies included such patients. Only one study (PAOLA-1) used bevacizumab with PARPi as maintenance, while the other three studies used single-agent PARPi. One study (VELIA) used PARPi with chemotherapy followed by maintenance, while the other three studies started PARPi maintenance after the end of chemotherapy. One study (PRIMA) used PARPi maintenance for 3 years, while the other studies used it for 2 years. Among statistical considerations, one study (VELIA) randomized patients before the beginning of chemotherapy, while the other studies randomized at the end of standard first-line treatment. Further, the included studies have varying follow-up durations, affecting data maturity variably. As stated in the Results section, the hazard ratios obtained from extracted data are not identical to the reported hazard ratios because the latter were ‘adjusted’ for covariates, which was not possible with our methodology. For example, PFS analysis in the PRIMA trial was performed using a one-sided log-rank test, stratified for best response during the first platinum regimen (complete response or partial response), high-risk characteristics (stage III with neoadjuvant treatment, stage III with adjuvant/first-line treatment and suboptimal cytoreduction, or stage IV), intraperitoneal or intravenous first-line platinum therapy, and geographic region. The reported PFS HR of 0.68 (0.49-0.94) in the HRD-negative population is adjusted for these factors, while the estimated PFS HR of 0.73 (0.53-1.01) is unadjusted. However, the use of unadjusted analysis is unlikely to substantially change the conclusions of the combined meta-analyzed population. Finally, although highly accurate, extracted individual survival data are not identical to original trial data and there could be minor deviations in the results compared with an IPD meta-analysis that uses original data.

In summary, this meta-analysis suggests that maintenance treatment with a PARPi after standard first-line treatment in newly diagnosed epithelial ovarian cancer results in substantial and clinically meaningful benefit in PFS among patients with germline and/or tumor BRCA mutation and/or homologous recombination-deficient tumors. This treatment strategy should be a standard of care in such patients. There is a lack of meaningful PFS benefit in patients without tumor HRD, and, given the significantly higher risk of toxicity with PARPis, this treatment is unlikely to be useful in such patients.
