# Supplementary material for: Maintenance therapy with a poly(ADP-ribose) polymerase inhibitor in patients with newly diagnosed advanced epithelial ovarian cancer: individual patient data and trial-level meta-analysis
Source: ESMO Open. 2022 Aug 22;7(5):100558. doi: 10.1016/j.esmoop.2022.100558 (PMC9588903; doi:10.1016/j.esmoop.2022.100558)

**SUPPLEMENTARY APPENDIX**

**Section I: Supplementary Methods**

Detailed methodology of individual patient data extraction and reconstruction of survival curves

**Section II: Supplementary Results**

**Supplementary Tables**

**Table S1:** Inclusion and exclusion criteria based on PICOS model

**Table S2:** Baseline clinical characteristics of whole meta-analyzed population from four trials

**Table S3:** Definition of survival endpoints in included trials

**Table S4:** Quality of evidence

**Table S5:** Estimated and reported events, hazard ratios and median PFS, by trial and treatment group

**Table S6:** Estimated progression-free survival at various time points using individual patient data from 3 RCT (excluding the trial by Moore, et al)

**Table S7:** Health-related quality-of-life outcomes

**Supplementary Figures**

**Figure S1:** PRISMA flow chart

**Figure S2:** Risk of bias in included trials

**Figure S3:** Progression-free survival comparing PARP inhibitor versus placebo using published estimates

**Figure S4:** Progression-free survival comparing PARPi versus placebo in biological subgroups: Forest plots of published estimates from 4 RCT.

(S4-A): Germline and/or tumor BRCA mutated

(S4-B): HRD positive including BRCA mutated

(S4-C): HRD positive excluding BRCA mutated

(S4-D): HRD negative

**Figure S5:** Progression-free survival comparing PARPi versus placebo in patient subgroups: Forest plots of published data from RCT.

(S5-A): Age

(S5-B): Eastern Cooperative Oncology Group performance status

(S5-C): Stage

(S5-D): Response to chemotherapy

(S5-E): Residual disease after surgery

**Figure S6:** Time-to-First-Subsequent-Therapy (TFST) comparing PARPi versus placebo: Forest plot of published estimates from 2 RCT

**Figure S7:** Progression-free survival two (PFS2) comparing PARPi versus placebo: Forest plot of published estimates from 2 RCT

**Figure S8:** Extracted events and reconstructed progression-free survival curves of PAOLA-1 trial (whole population) by Coquard IR, et al

(S8-A): Extracted events

(S8-B): Reconstructed PFS curves

**Figure S9:** Extracted events and reconstructed progression-free survival curves of PAOLA-1 trial (HRD positive subgroup, including BRCA mutated) by Coquard IR, et al

(S9-A): Extracted events

(S9-B): Reconstructed PFS curves

**Figure S10:** Extracted events and reconstructed progression-free survival curves of PAOLA-1 trial (HRD positive subgroup excluding BRCA mutated) by Coquard IR, et al

(S10-A): Extracted events

(S10-B): Reconstructed PFS curves

**Figure S11:** Extracted events and reconstructed progression-free survival curves of PAOLA-1 trial (HRD negative subgroup excluding BRCA mutated) by Coquard IR, et al

(A11-A): Extracted events

(A11-B): Reconstructed PFS curves

**Figure S12:** Extracted events and reconstructed progression-free survival curves of PAOLA-1 trial (tumor BRCA mutated subgroup) by Coquard IR, et al

(S12-A): Extracted events

(S12-B): Reconstructed PFS curves

**Figure S13:** Extracted events and reconstructed progression-free survival curves of PRIMA trial (whole population) by Martin AG, et al

(S13-A): Extracted events

(S13-B): Reconstructed PFS curves

**Figure S14:** Extracted events and reconstructed progression-free survival curves of PRIMA trial (HRD positive subgroup including BRCA mutated) by Martin AG, et al

(S14-A): Extracted events

(S14-B): Reconstructed PFS curves

**Figure S15:** Extracted events and reconstructed progression-free survival curves of PRIMA trial (HRD positive subgroup, excluding BRCA mutated) by Martin AG, et al

(S15A): Extracted events

(S15-B): Reconstructed PFS curves

**Figure S16:** Extracted events and reconstructed progression-free survival curves of PRIMA trial (HRD negative subgroup) by Martin AG, et al

(S16-A): Extracted events

(S16-B): Reconstructed PFS curves

**Figure S17:** Extracted events and reconstructed progression-free survival curves of PRIMA trial (BRCA 1 mutated subgroup) by Martin AG, et al

(S17-A): Extracted events

(S17-B): Reconstructed PFS curves

**Figure S18:** Extracted events and reconstructed progression-free survival curves of PRIMA trial (BRCA 2 mutated subgroup) by Martin AG, et al

(S18A): Extracted events

(S18-B): Reconstructed PFS curves

**Figure S19:** Extracted events and reconstructed progression-free survival curves of PRIMA trial (combined BRCA ½ mutated population) by Martin AG, et al

(S19-A): Extracted events

(S19-B): Reconstructed PFS curves

**Figure S20:** Extracted events and reconstructed progression-free survival curves of VELIA trial (whole population) by Coleman RL, et al

(S20-A): Extracted events

(S20-B): Reconstructed PFS curves

**Figure S21:** Extracted events and reconstructed progression-free survival curves of VELIA trial HRD positive subgroup, including BRCA mutated) by Coleman RL, et al

(S21-A): Extracted events

(S21-B): Reconstructed PFS curves

**Figure S22:** Extracted events and reconstructed progression-free survival curves of VELIA trial (HRD negative subgroup) by Coleman RL, et al

(S22-A): Extracted events

(S22-B): Reconstructed PFS curves

**Figure S23:** Extracted events and reconstructed progression-free survival curves of VELIA trial (BRCA1/2 mutated subgroup) by Coleman RL, et al

(S23-A): Extracted events

(S23-B): Reconstructed PFS curves

**Figure S24:** Extracted events and reconstructed progression-free survival curves of SOLO-1 trial by Moore K, et al (updated data)

(S24-A): Extracted events

(S24-B): Reconstructed PFS curves

**SECTION I: SUPPLEMENTARY METHODS**

**Detailed methodology of individual patient data extraction and reconstruction of survival curves**

The WebPlotDigitizer software was used to extract data from published PFS Kaplan-Meier curves. This data was extracted manually for each trial through an iteration process until the extracted number of PFS events matched closely with the published ones at each time point. Data extraction quality was evaluated based on estimated and published PFS durations by study arm, hazard ratios with their 95% confidence intervals, year-wise event numbers by study arm, and duration of follow-up in each trial. Using this individual patient level extracted data and published numbers at risk we reconstructed PFS curves for each study using the STATA command ipdfc, published by Wei et al.

Individual patient data for whole study populations from three eligible studies (PRIMA, PAOLA-1, and VELIA) were combined and PFS Kaplan-Meier curves were generated by study arm (PARPi vs placebo). The SOLO1 study included only patients with germline *BRCA1* or *BRCA2* mutation, therefore data from this study was not combined with whole study populations of the other three studies, which included patients with and without *BRCA* mutations. Data from SOLO1 trial was combined with the *BRCA* mutated subgroups of other three studies, as described in the section on subgroup analyses. The forest plot for PFS for the combined whole population was constructed using the extracted data of these three studies. One arm of VELIA study used PARP inhibitor only during the period of first-line chemotherapy (n=383), therefore data from this arm was excluded from the analysis. Patients with unknown HRD status were also excluded from the subgroup analysis as shown below.

| *Trial* | *No of patients excluded from the subgroup analysis with HRD unknown status*  *PARPi arm Placebo arm* | |
| --- | --- | --- |
| *POALA-1* | *90* | *52* |
| *PRIMA* | *71* | *40* |

Additionally, we also estimated the number of events and proportion (with 95% CI) of patients surviving progression-free at each time point (1-year, 2-year, and 3-year) from the combined IPD PFS curves.

The included studies have variably reported results by germline and/or somatic *BRCA1* or *BRCA2* mutation status. Therefore, for this analysis, we defined *BRCA* mutation positive status as the presence of pathogenic or likely pathogenic variants in *BRCA1* or *BRCA2* genes in germline and/or tumor tissue. We extracted individual patient data from published or presented PFS curves for the following patient subgroups defined by *BRCA* mutation and HRD status: with presence of *BRCA* mutation (4 trials), with HRD positive tumors including those with presence of *BRCA* mutation (3 trials), with HRD positive tumors excluding those with presence of *BRCA* mutation (2 trials), and those with HRD negative tumors (3 trials). Individual patient data by study arm were combined for these subgroups, with generation of PFS Kaplan-Meier curves.

**SECTION II: SUPPLEMENTARY RESULTS**

**Table S1:** Study inclusion and exclusion criteria based on PICOS model

| Parameter | Inclusion criteria | Exclusion criteria |
| --- | --- | --- |
| Patients | Patients with stage III or IV epithelial ovarian, primary peritoneal or fallopian tube cancer, after completion of first line platinum-based therapy | Patients with recurrent disease |
| Interventions | PARP inhibitors | Immunotherapy, cancer vaccines |
| Comparator | Placebo | Not applicable |
| Outcomes | PFS, toxicity | Outcome on HRD unknown status |
| Study design | Phase III randomized controlled trials | Retrospective studies, single arm prospective trials |

**Table S2: Definition of survival end points in included trials**

| **Study** | **PFS calculated from DOR** | **Time of Randomization** | **PFS** | **TFST** | **PFS2** |
| --- | --- | --- | --- | --- | --- |
| Coquard IR  (PAOLA-1) | Yes | After achieving PR/CR to platinum-based therapy | Disease progression on imaging or death due to any cause | Time from the DOR to date of the first subsequent anti- cancer therapy or death due to any cause | Time from the DOR to date of second progression * or death due to any cause |
| Martin AG  (PRIMA) | Yes | After achieving PR/CR to platinum-based therapy | Disease progression on imaging or death due to any cause | Time from the DOR to date of the first subsequent anti- cancer therapy or death due to any cause | Time from the DOR to date of second progression * or death due to any cause |
| Coleman RL  (VELIA) | Yes | At the beginning of chemotherapy | Disease progression on imaging or death due to any cause | Time from the DOR to date of the first subsequent anti- cancer therapy or death due to any cause | Time from the DOR to date of second progression * or death due to any cause |
| Moore K  (SOLO1) | Yes | After achieving PR/CR to platinum-based therapy | Disease progression on imaging or death due to any cause | Time from the DOR to date of the first subsequent anti- cancer therapy or death due to any cause | Time from the DOR to date of second progression * or death due to any cause |

Abbreviations: DOR, date of randomization; PFS, progression free survival; TSFT, time to first subsequent therapy; PFS2, second progression free survival

*The date of second progression was defined according to local standard clinical practice and may involve any of; objective radiological, CA-125 or symptomatic progression or death

**Table S3:** Baseline clinical characteristics of whole meta-analyzed population from four trials

| **Characteristics** | **Experimental arm (PARPi)**  **N (%)**  **1666** | **Control arm**  **(placebo)**  **N (%)**  **1021** | **Total population**  **N (%)**  **2687** |
| --- | --- | --- | --- |
| Age  <65  >65 | 1082 (65%)  584 (35%) | 674 (66%)  347 (34%) | 1756 (65%)  931 (35%) |
| ECOG score  0  1 | 1139 (69%)  516 (31%) | 694 (68%)  318 (32%) | 1833 (68%)  834 (32%) |
| Stage  III  IV | 1211 (73%)  455 (27%) | 742 (73%)  279 (27%) | 1953 (73%)  734 (27%) |
| Receipt of neoadjuvant chemotherapy  Yes  No | 794 (48%)  858 (52%) | 458 (45%)  563 (55%) | 1252 (47%)  1421 (53%) |
| Response after first line therapy  CR  PR | 915 (55%)  526 (32%) | 639 (63%)  360 (35%) | 1554 (58%)  886 (33%) |
| Deleterious *BRCA* mutation  *gBRCA* mutated  *tBRCA* mutated  *g/tBRCA* mutated | 440 (26%)  185 (11%)  152 (9%) | 194 (19%)  109 (10%)  71 (7%) | 634 (23%)  294 (11%)  223 (8%) |
| HRD status  HRD positive including BRCA mutated  HRD positive excluding BRCA mutated  HRD negative  HRD unknown | 621 (37%)  192 (12%)  486 (29%)  161 (9%) | 410 (40%)  110 (11%)  289 (28%)  92 (9%) | 1031 (39%)  302 (11%)  775 (29%)  253 (9%) |

Abbreviations: N, number of patients; PARPi, PARP inhibitor; HRD, homologous recombination deficiency.

*gBRCA, germline BRCA mutated; tBRCA, tumor BRCA mutated; g/tBRCA, germline and or tumor BRCA mutated

**Table S4: Quality of Evidence**

| Quality assessment | | | | | | | No of patients | | Effect | | Quality | Importance |
| --- | --- | --- | --- | --- | --- | --- | --- | --- | --- | --- | --- | --- |
|  |  |  |  |  |  |  |  |  |  |  |  |  |
| No of studies | Design | Risk of bias | Inconsistency | Indirectness | Imprecision | Other considerations | Progression Free Survival | Control | Relative (95% CI) | Absolute |  |  |
| PFS comparison | | | | | | | | | | | | |
| 3 | RCTs | none | none | none | none | none | 703/1406  (50%) | 586/890  (65.8%) | HR 0.63 (0.56 to 0.71) | 167 fewer per 1000 (from 125 fewer to 206 fewer) |   High | Important |
|  |  |  |  |  |  |  |  | 63.2% |  | 165 fewer per 1000 (from 124 fewer to 203 fewer) |  |  |

RCTs – Randomized controlled trials

|  |  | **PARPi (events)** | | | **Placebo (events)** | | | **HR (95 % CI)** | | **PARPi (median PFS)**  **(months)** | | **Placebo (median PFS)**  **(months)** | |
| --- | --- | --- | --- | --- | --- | --- | --- | --- | --- | --- | --- | --- | --- |
| **Endpoint** | **Study** | **Estimated (N)** | **Reported (N)** | **diff** | **Estimated (N)** | **Reported (N)** | **diff** | **Estimated *** | **Reported** | **Estimated** | **Reported** | **Estimated** | **Reported** |
| PFS | Coleman RL (VELIA) | 189 | 191 | -2 | 236 | 237 | -1 | 0.69  (0.57- 0.83) | 0.68  (0.56-0.83)^ | 23.6 (20.16-26.74) | 23.5  (19.3-26.3) | 17.55 (15.25-19.64) | 17.3 (15.1-19.1) |
|  | Coquard IR  (PAOLA-1) | 280 | 280 | 0 | 194 | 194 | 0 | 0.59  (0.49-0.71) | 0.59  (0.49-0.72)^@^ | 22.31  (21.69-24.09) | 22.1 | 16.78  (15.55-18.68) | 16.6 |
|  | Martin AG  (PRIMA) | 233 | 232 | +1 | 156 | 155 | +1 | 0.60  (0.49-0.74) | 0.62  (0.50-0.76)^$^ | 13.73  (11.36-14.80) | 13.8 (11.5-14.9) | 8.07  (7.20- 8.39) | 8.2  (7.3-8.5) |
|  | Moore K  (SOLO-1) | 118 | 118 | 0 | 100 | 100 | 0 | 0.37  (0.28-0.48) | 0.33  (0.25- 0.43)^#^ | 55.85  (42 – NR) | 56 | 14  (11.18-18) | 13.8 |

**Table S5:** **Estimated and reported events, hazard ratios and median PFS, by trial and treatment group**

Abbreviations: N, number of patients; PFS, progression free survival; PARPi, PARP inhibitor; HR, hazard rate

*All estimated HR were unadjusted

#HR adjusted according to response to first line treatment

^HR adjusted according to disease stage, residual disease status, choice of paclitaxel regimen and BRCA mutation status

^@^ HR adjusted according to response to first line treatment and germline BRCA status

$ HR adjusted according to response to first line treatment, administration of neoadjuvant therapy and HRD status

**Table S6: Estimated progression-free survival at various time points using individual patient data from three RCT**

|  |  | **Estimated progression -free survival** | | | **Reported progression-free survival (95% CI)** | |
| --- | --- | --- | --- | --- | --- | --- |
| Time | Study | PARPi (%, 95% CI) | Placebo (%, 95% CI) | Difference (%, 95% CI) | PARPi | Placebo |
| 1 year | Coleman RL  (VELIA ) | 78.81 (73.99-82.85) | 70.99 (65.82 -75.52) | 7.82 (2.90-15.97) | 84%*  (80-88) | 77%*  (72-81) |
|  | Coquard IR  (PAOLA-1) | 78.07 (74.25-81.31) | 65.88 (59.81-71.25) | 12.19 (6.47-19.83) |  |  |
|  | Martin AG  (PRIMA) | 53.60 (48.65-58.3) | 34.63 (28.36- 40.96) | 18.97 (11.76-27.50) | 53% | 35% |
|  | Combined | 70.23 (67.63-72.66) | 59.52 (56.08-62.78) | 10.71 (6.99-15.33)  P< 0.001 |  |  |
| 2 year | Coleman RL  (VELIA ) | 47.99 (42.29-53.45) | 34.18 (29.01-39.41) | 13.81 (7.29-22.38) | 48%  (42-53) | 34%  (29-39) |
|  | Coquard IR  (PAOLA-1) | 45.99 (41.27-50.57) | 27.86 (22.28-33.70) | 18.13 (11.43-26.06) |  |  |
|  | Martin AG  (PRIMA) | 31.67 (24.28 -39.30) | 23.59 (15.5-32.60) | 8.08 (1.20-23.94) |  |  |
|  | Combined | 41.91 (38.82-44.97) | 27.94 (24.64-31.33) | 13.97 (9.80-18.86)  P< 0.001 |  |  |
| 3 year | Coleman RL  (VELIA ) | 32.41 (25.34-39.66) | 20.11(14.23-26.74) | 12.30 (4.79-23.58) |  |  |
|  | Coquard IR  (PAOLA-1) | 34.88 (27.69-42.15) | 18.38 (12.90-24.63) | 16.50 (8.47-26.85) |  |  |
|  | Martin AG  (PRIMA) | - | - | - |  |  |
|  | Combined | 30.43 (26.20-34.77) | 16.90 (13.06-21.18) | 13.53 (8.30-20.04)  P< 0.001 |  |  |

Abbreviations: PARPi, PARP inhibitor; *PFS reported at 10 months

**Table S7: Health-related Quality of life outcomes**

| Study | QOL instrument | QOL outcome measure | Significant difference | QOL  Result | Compliance rate to QOL index |
| --- | --- | --- | --- | --- | --- |
| Moore K  (SOLO1 ) | Trial outcome index (TOI)  (score ranges from 0-100)  summary index of physical and functional well-being and key ovarian cancer symptoms derived from the FACT-O questionnaire | Change in TOI score from baseline | Difference of 10 points or more | Mean change from baseline 0.30 points (95 %CI, -0.72 to 1.32) in PARPi group  Mean change from baseline 3.30 points (95 %CI,1.84 to 4.76) in placebo group  Between group difference in change, -3.00 points (95%CI, −4.78 to −1.22) | >80 % |
| Martin AG  (PRIMA) | Patient reported outcome (PRO) assessed by FOSI,  EQ-5D-5L, EORTC-QLQ-C30/OV28 | Change in PRO from baseline | - | No difference in Health-related quality of score between PARPi and placebo | >80% |
| Coleman RL  (VELIA) | National Comprehensive Cancer Net- work Functional Assessment of Cancer Therapy Ovarian Symptom Index–18 (NFOSI-18) | Change in NFOSI-18 score from baseline | Difference of 3 point or more | Difference in mean change from baseline in score between treatment group, 0.0 to 2.1 | >80%  86% of patients had >90% compliance |
| Coquard IR  (PAOLA-1) | EORTC QLQ-C30  (Score ranges from 0- 100) | Change in EORTC QLQ-C30  from baseline | Difference of 10 points or more | Mean change from baseline, −1.33 points (95%CI, −2.47 to −0.19) in the PARPi group  Mean change from baseline, −2.89 points (95%CI, −4.52 to −1.26) in the placebo group  Between group difference in change, 1.56 points (95%CI, −0.42 to 3.55) | >90% |

Abbreviations: FACT-O, Functional Assessment of Cancer Therapy–Ovarian Cancer; FOSI, Functional Assessment of Cancer Therapy-Ovarian Symptom Index; EORTC-QLQ-C30, European Organization for Research and Treatment of Cancer Quality of Life Questionnaire; EQ-5D-5L, European Quality of Life five-dimension, five-level questionnaire; EORTC-QLQ-OV28, EORTC Quality of Life Questionnaire Ovarian Cancer module.

**Figure S1:** **PRISMA flow chart**

Conference proceedings
(n =18)

Ongoing trials - 7

Records identified through database searching (Pubmed, Cochrane, Embase)
(n =408)

Excluded articles [n- 276]

Review articles – 217

Guidelines on Rx- 1

Individual PARP inhibitor drug review - 28

Biomarkers for PARP inhibitors - 9

Toxicities of PARP inhibitor – 4

Resistance to PARP inhibitors -2

PK studies - 2

PARP inhibitors in other malignancy – 2

End points in ovarian cancer – 4

Ongoing trials - 7

Records after duplicates removed
(n =335)

Full-text articles assessed for eligibility
(n = 59)

Full-text articles excluded [n- 36]

Retrospective analysis – 3

RCT of maintenance PARPi in recurrent setting

Phase II – 6

Phase III-7

CEA (cost effective analysis) of PARPi - 6

Meta-analyses (recurrent setting) – 4

Network meta analysis comparing CT/PARPi/antiangiogenic therapies -2

Meta analysis in first line setting - 4

QOL studies with PARPi - 4

Articles/conference proceedings/abstracts included in qualitative synthesis

(n= 23)

23 publications corresponding to 4 studies included in quantitative synthesis
(n =4)

**Figure S2: Risk of bia**s


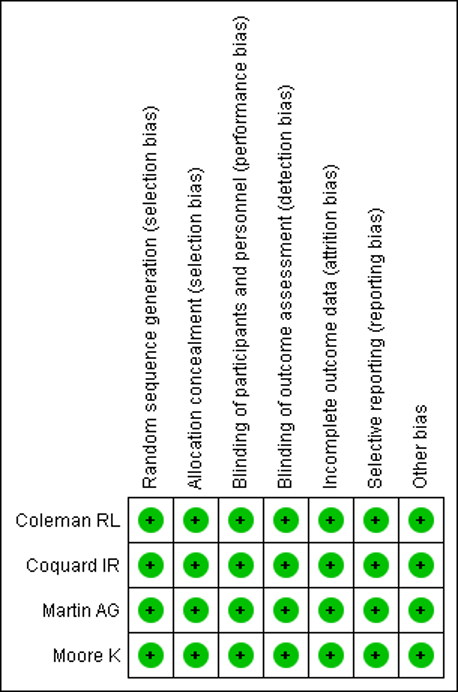


**Figure S3:** Progression free survival comparing PARP inhibitor versus placebo using published estimates from 3 RCT (excluding the trial by Moore, et al)


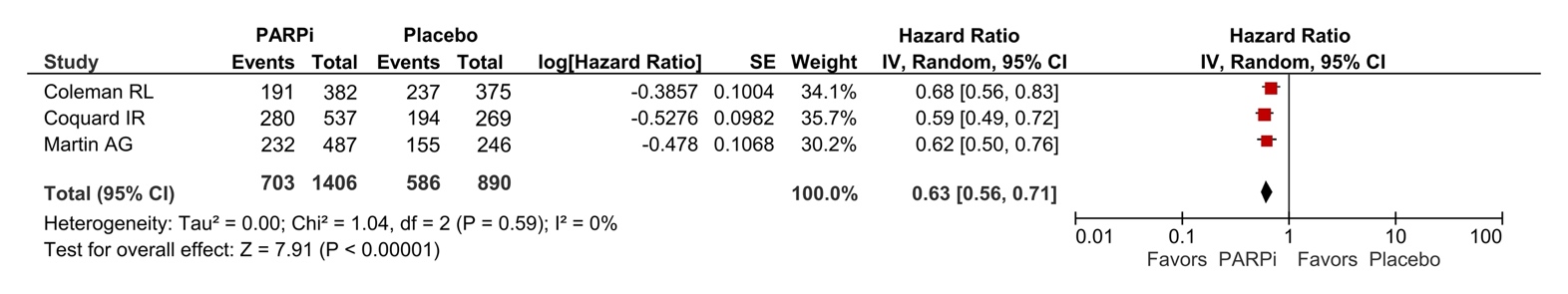


**Figure S4:** Progression-free survival comparing PARPi versus placebo in biological subgroups: Forest plots of published estimates from 4 RCT.

(S4-A): Germline and/or tumor BRCA mutated

(S4-B): HRD positive including BRCA mutated

(S4-C): HRD positive excluding BRCA mutated

(S4-D): HRD negative


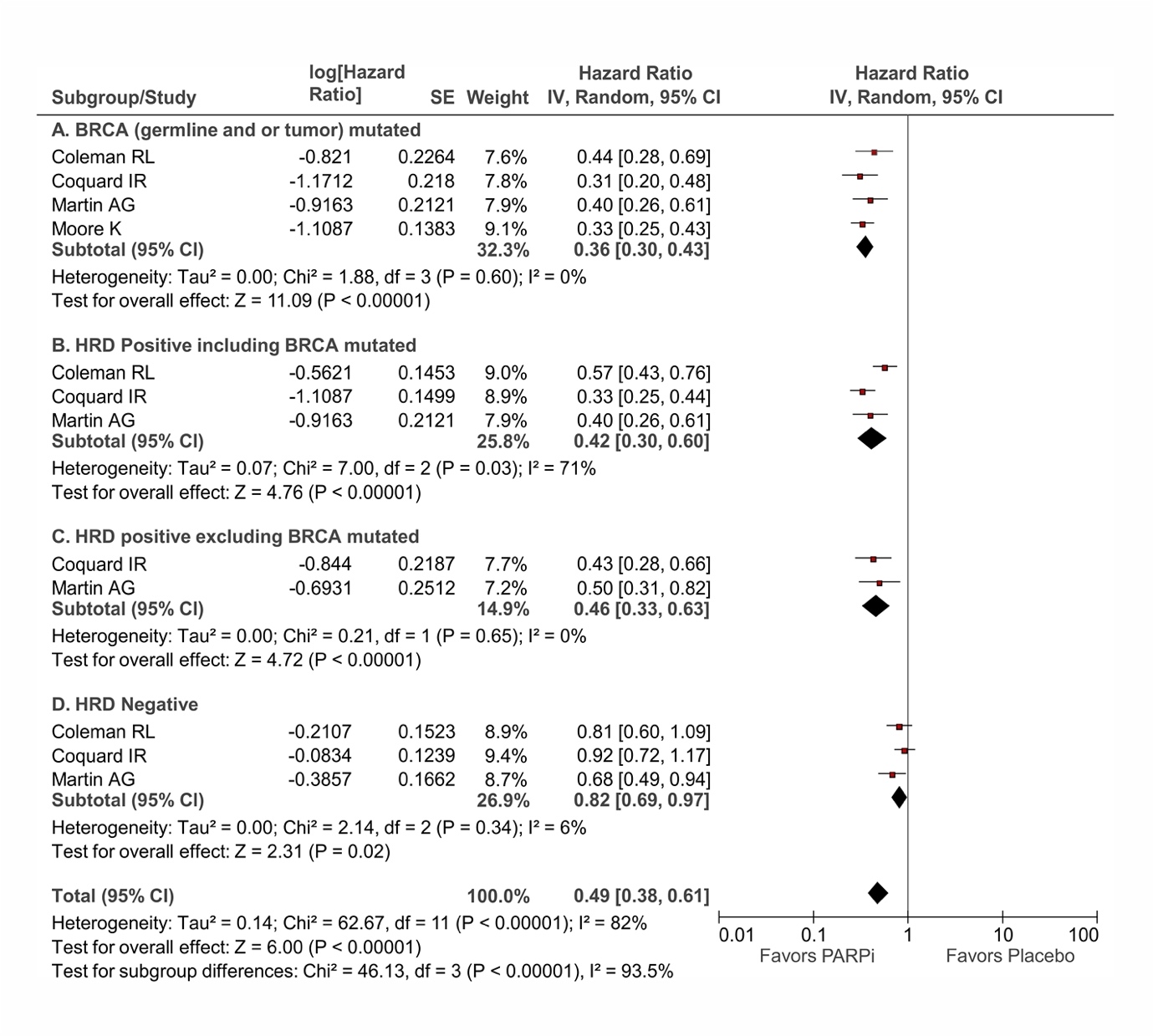


**Figure S5:** Progression-free survival comparing PARPi versus placebo in patient subgroups: Forest plots of published data from RCT.

(S5-A): Age

(S5-B): Eastern Cooperative Oncology Group (ECOG) performance status

(S5-C): Stage

(S5-D): Response to chemotherapy

(S5-E): Residual disease after surgery

**Figure S5-A**: Age


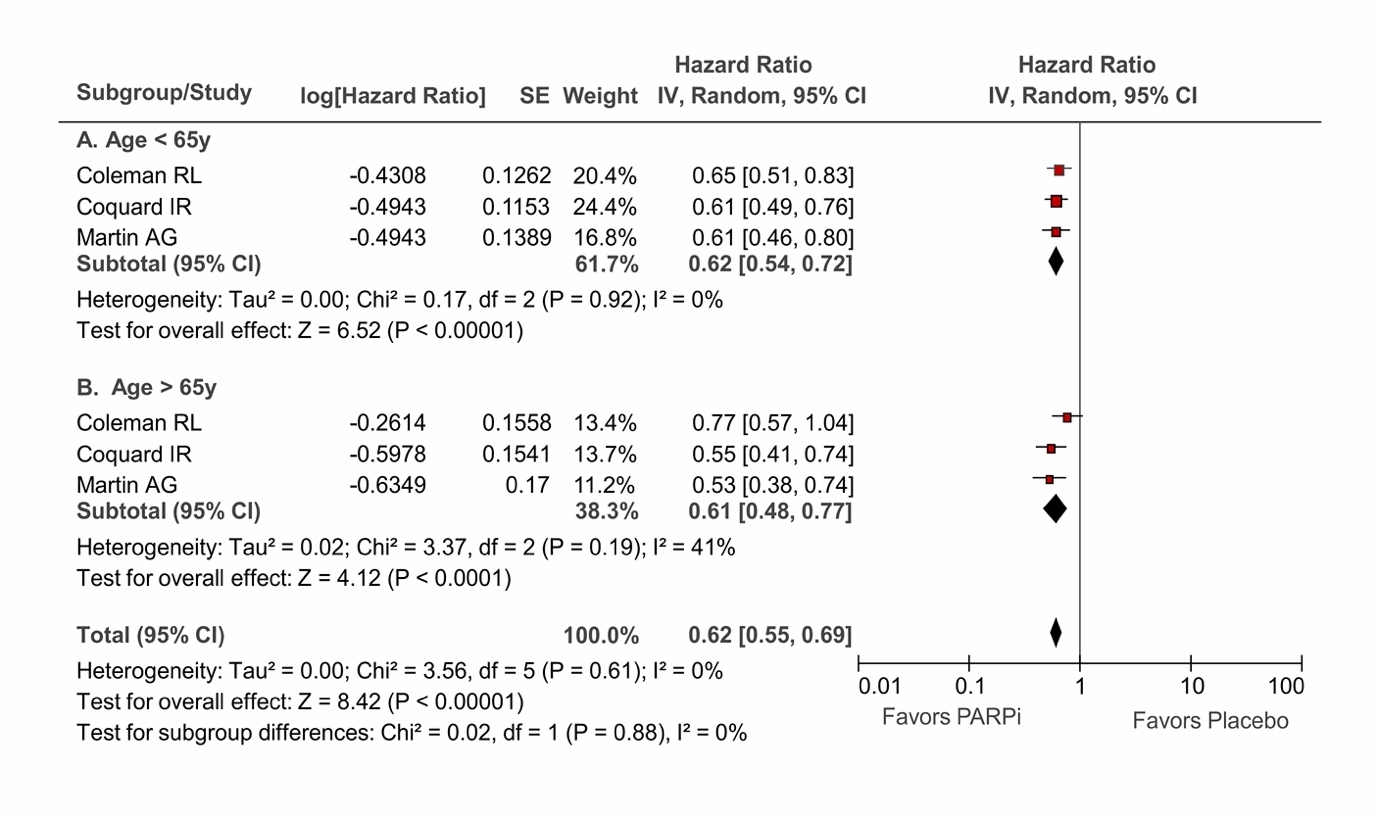


**Figure S5-B**: ECOG performance status


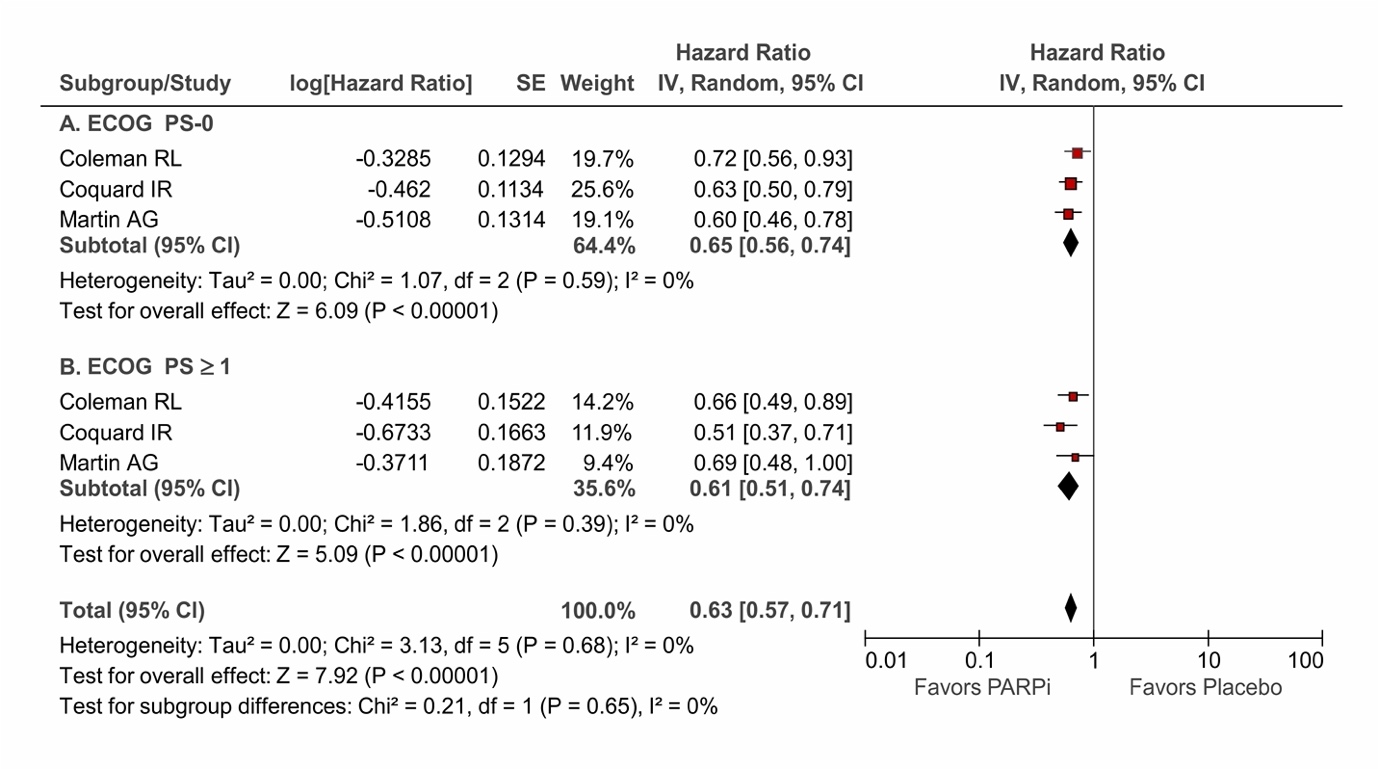


**Figure S5-C**: Stage


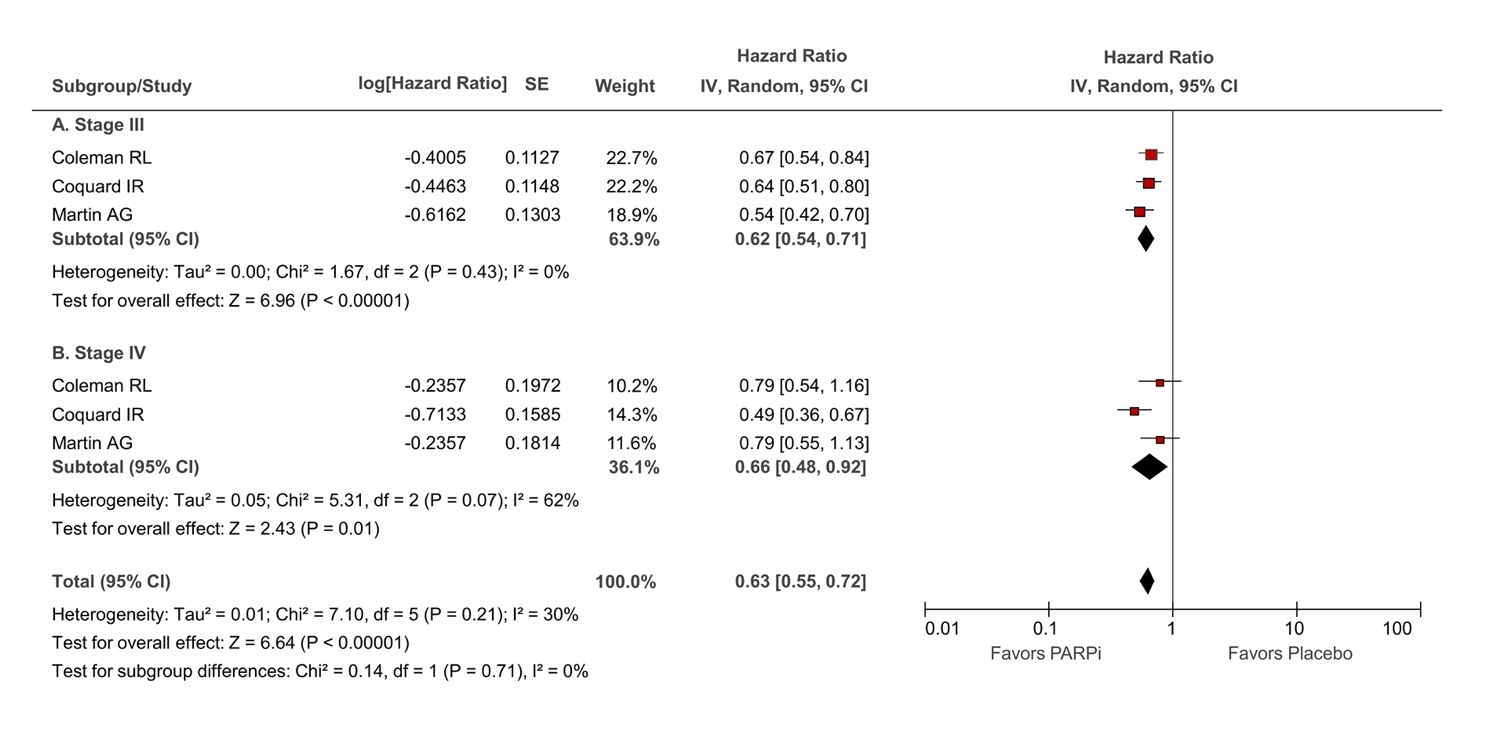


**Figure S5-D**: Response to chemotherapy


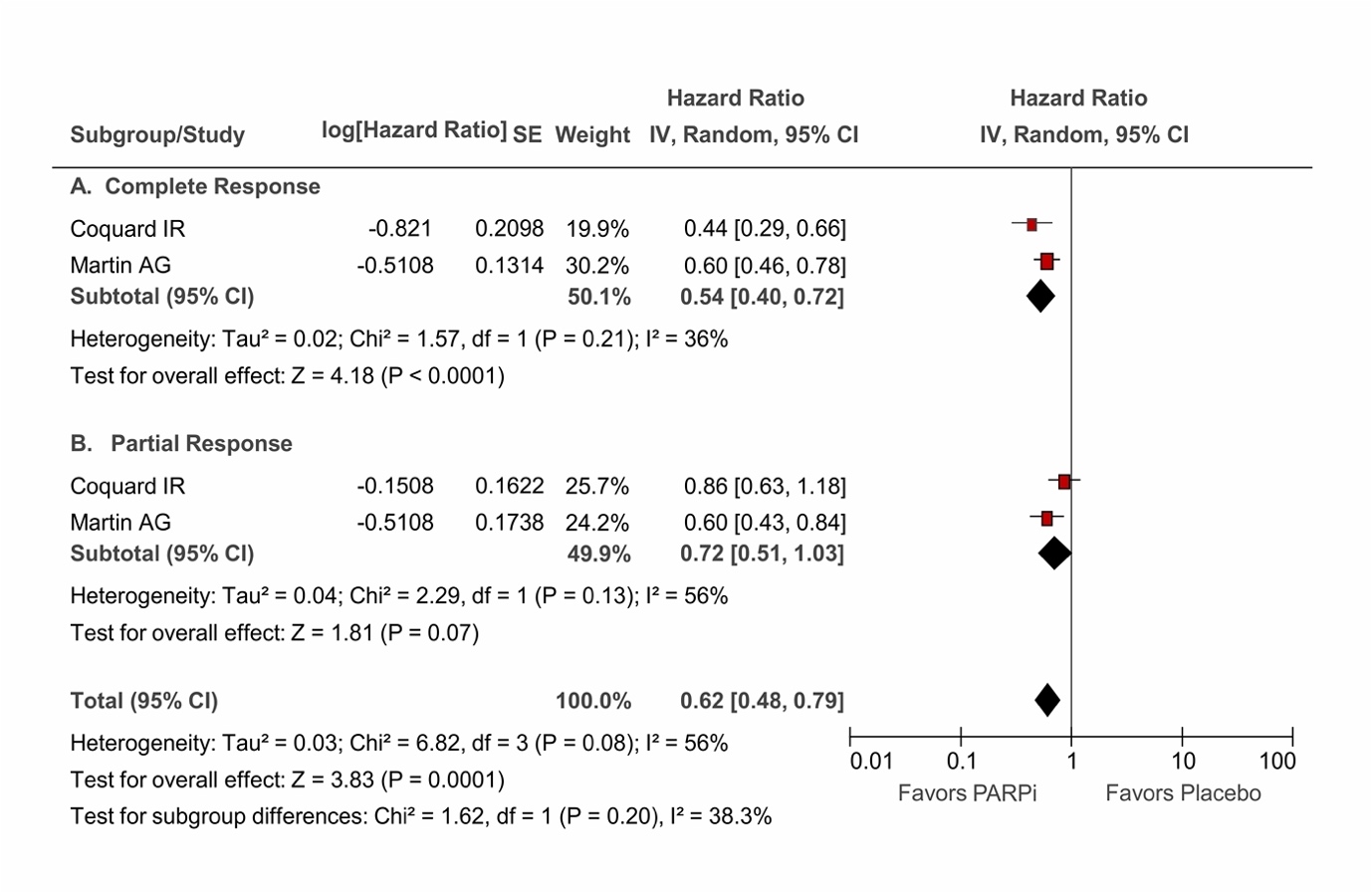


**Figure S5-E**: Residual disease post-surgery


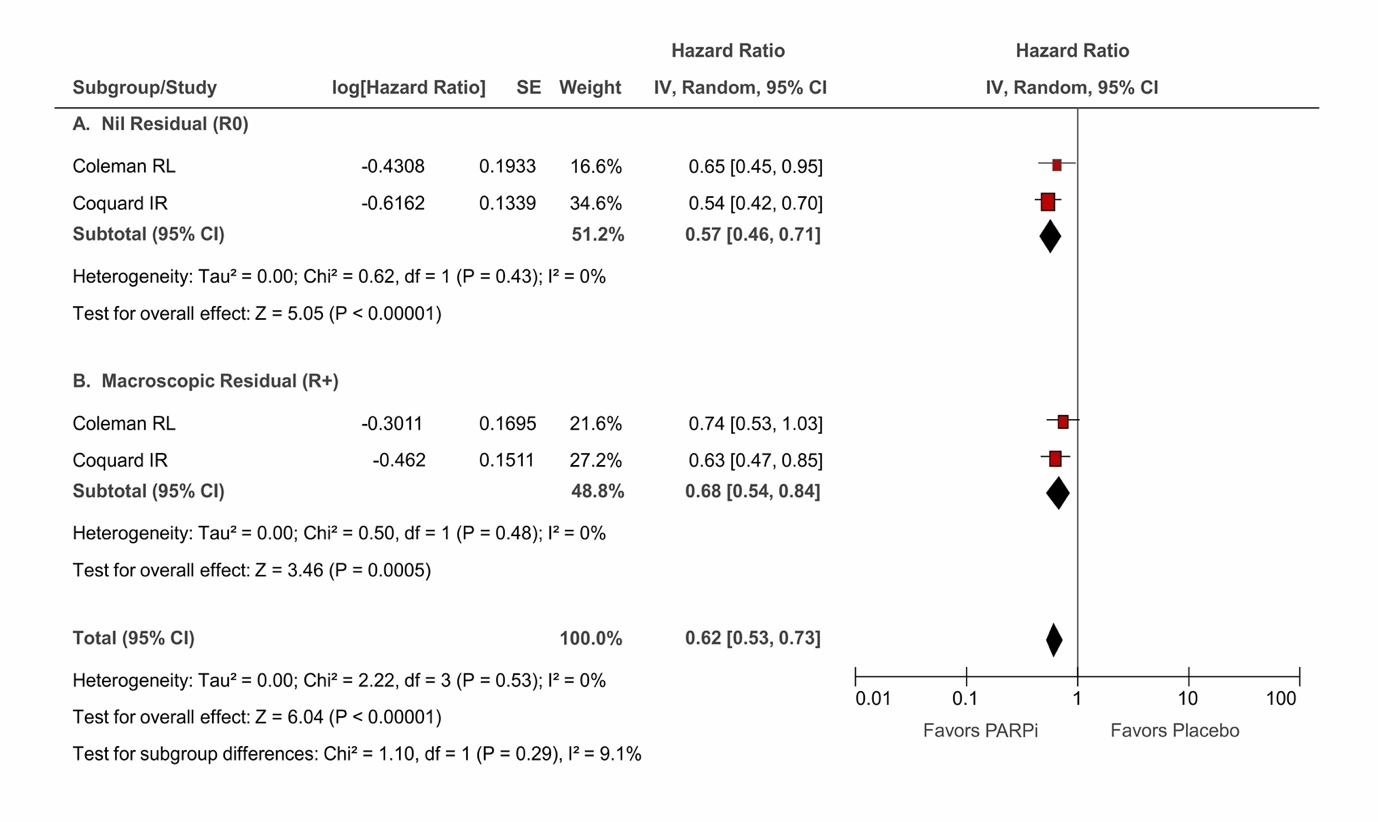


**Figure S6:** Time-to-First-Subsequent-Therapy (TFST) comparing PARPi versus placebo: Forest plot of published estimates from 2 RCT


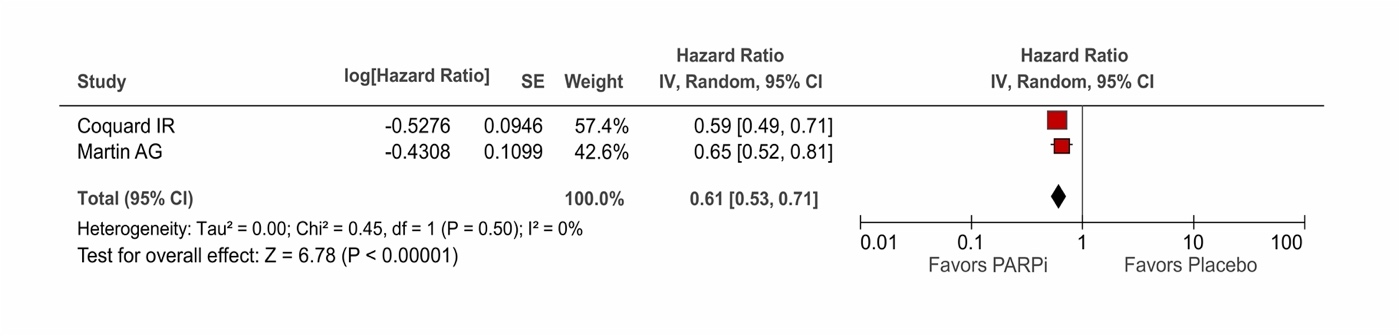


**Figure S7:** Progression-free survival two (PFS2) comparing PARPi versus placebo: Forest plot of published estimates from 2 RCT


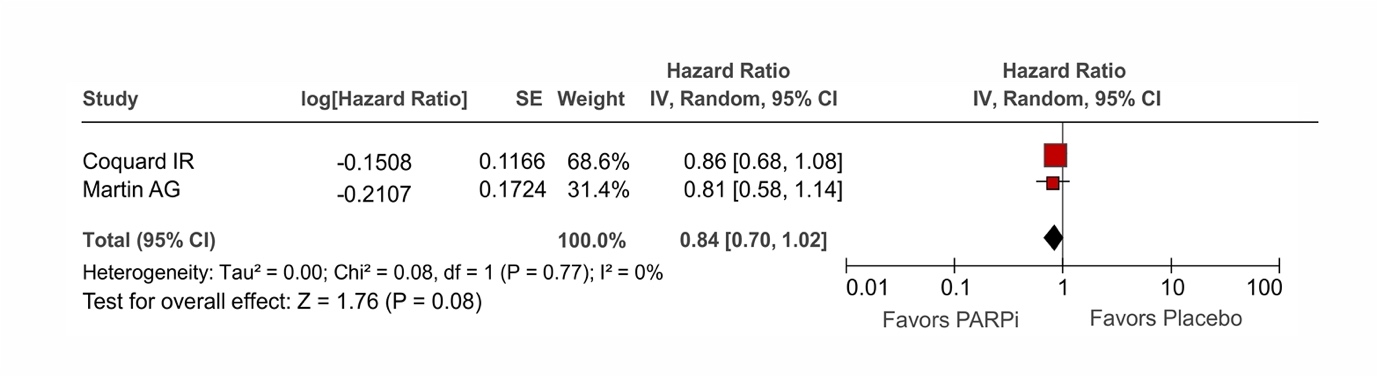


**Figure S8:** Extracted events and reconstructed progression-free survival curves of PAOLA-1 trial (whole population) by Coquard IR, et al

**Figure S8-A:** Extracted events

Events in PARPi arm – 280

Events in placebo arm – 194

**Figure S8-B:** reconstructed progression free survival curves


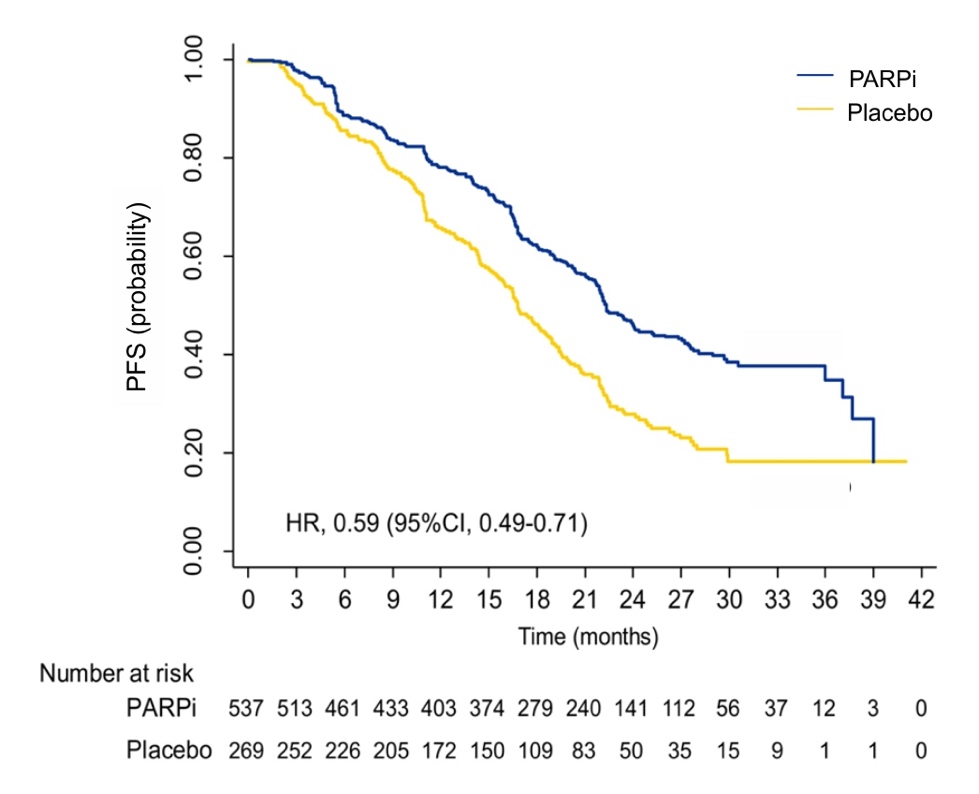


**Figure S9:** Extracted events and reconstructed progression-free survival curves of PAOLA-1 trial (HRD positive subgroup, including BRCA mutated) by Coquard IR, et al

**Figure S9-A:** Extracted events

**Figure S9-B:** Reconstructed progression-free survival curves


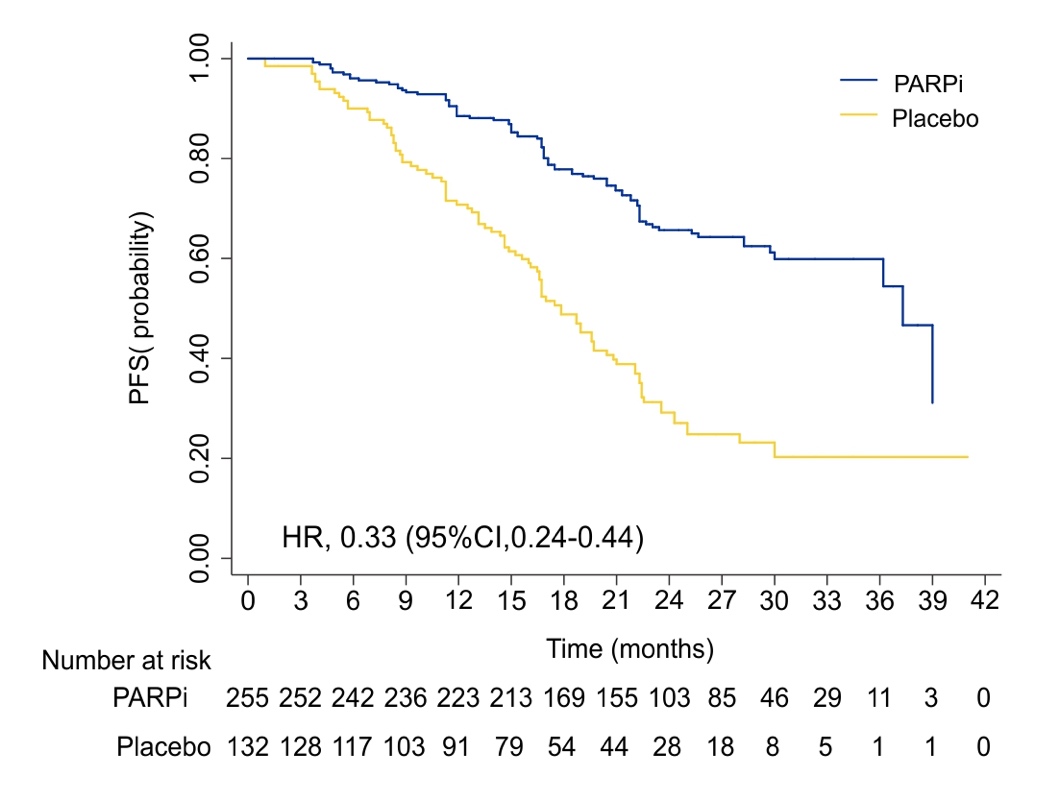


**Figure S10:** Extracted events and reconstructed progression-free survival curves of PAOLA-1 trial (HRD positive subgroup, excluding BRCA mutated) by Coquard IR, et al

**Figure S10-A:** Extracted events

**Figure S10-B:** Reconstructed progression-free survival curves


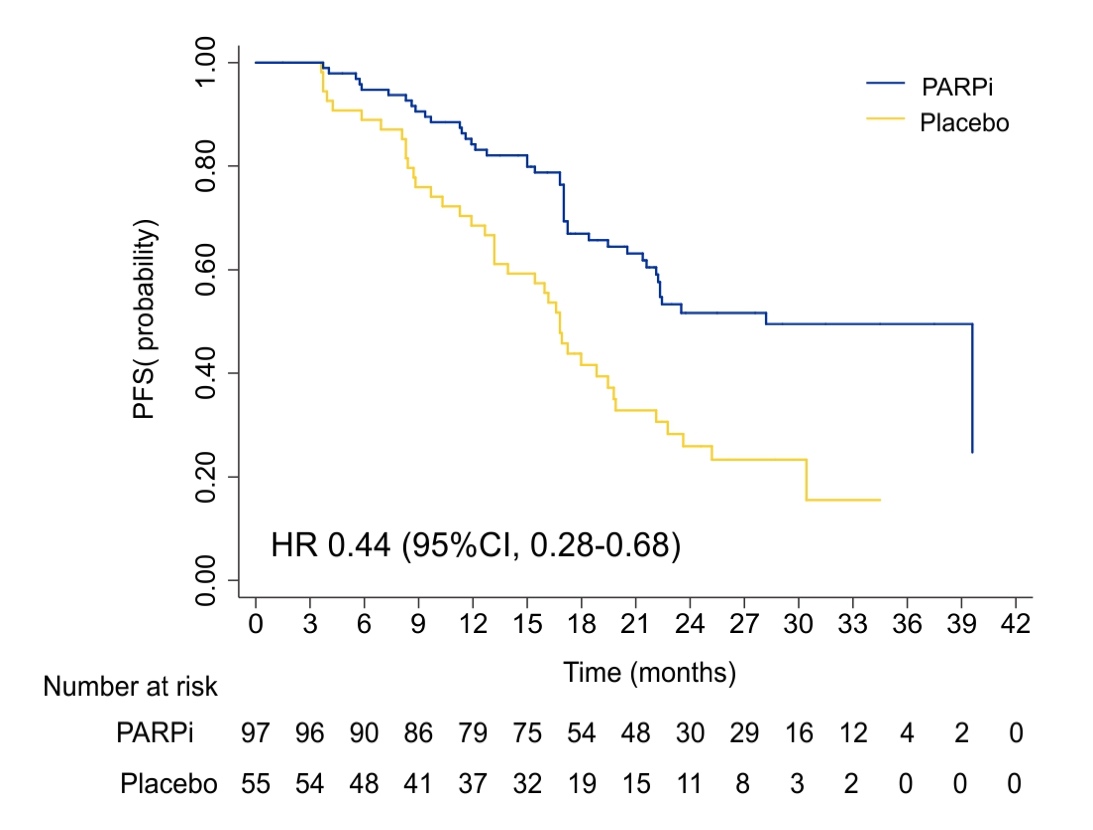


**Figure S11:** Extracted events and reconstructed progression-free survival curves of PAOLA-1 trial (HRD negative subgroup) by Coquard IR, et al

**Figure S11-A:** Extracted events

Events in PARPi arm – 194

Events in Placebo arm – 102

**Figure S11-B:** Reconstructed progression-free survival curves


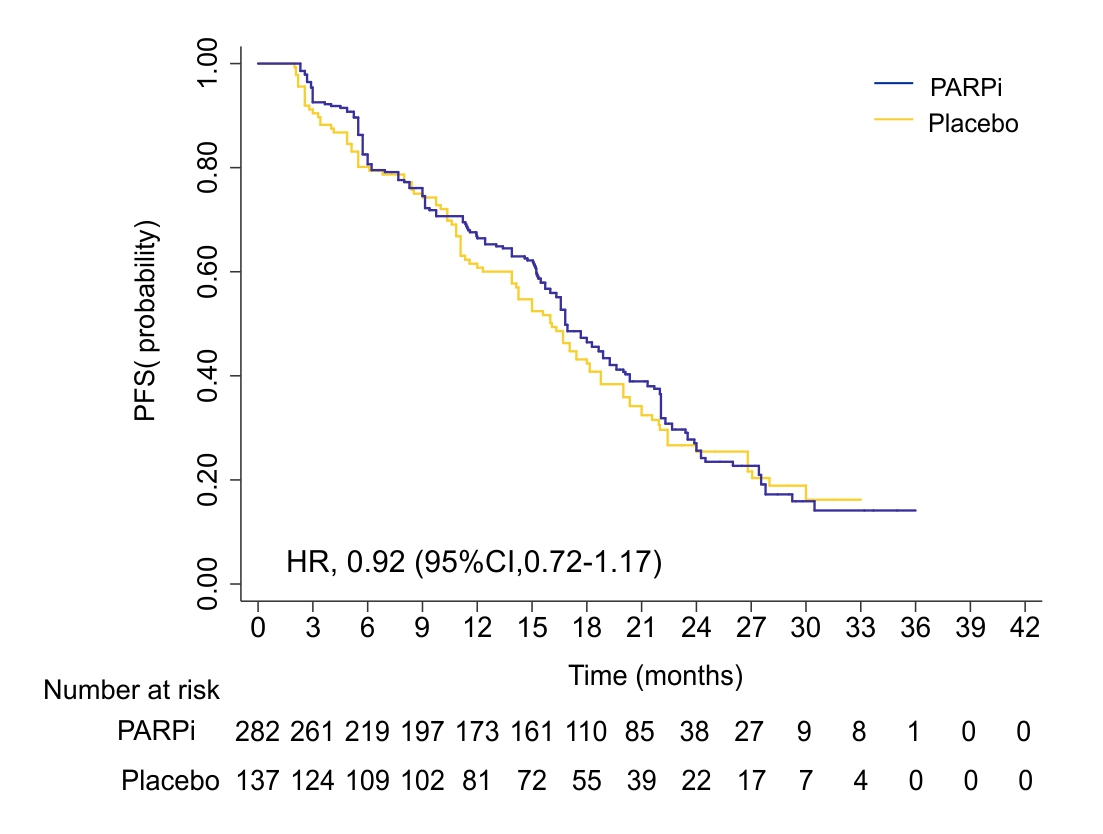


**Figure S12:** Extracted events and reconstructed progression-free survival curves of PAOLA-1 trial (tumor BRCA mutated subgroup) by Coquard IR, et al

**Figure S12-A:** Extracted events

**Figure S12-B:** Reconstructed progression-free survival curves


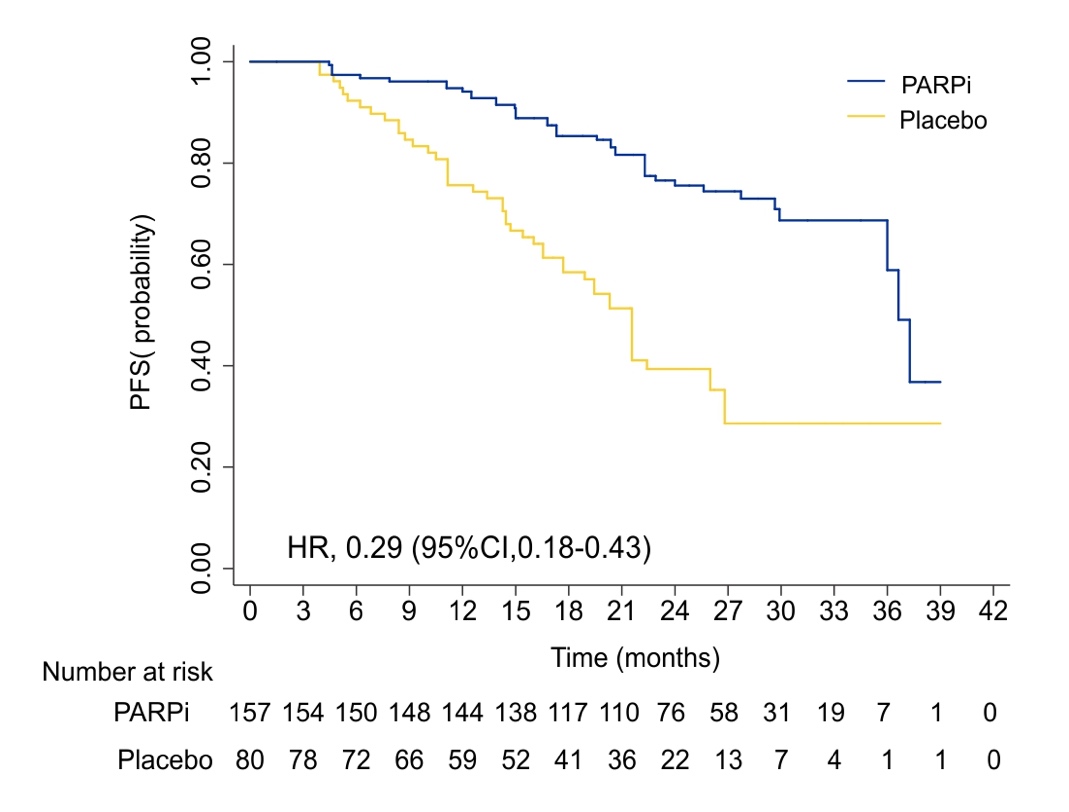


**Figure S13:** Extracted events and reconstructed progression-free survival curves of PRIMA trial (whole population) by Martin AG, et al

**Figure S13-A:** Extracted events

Events in PARPi arm – 233

Events in Placebo arm – 156

**Figure S13-B:** Reconstructed progression- free survival curves


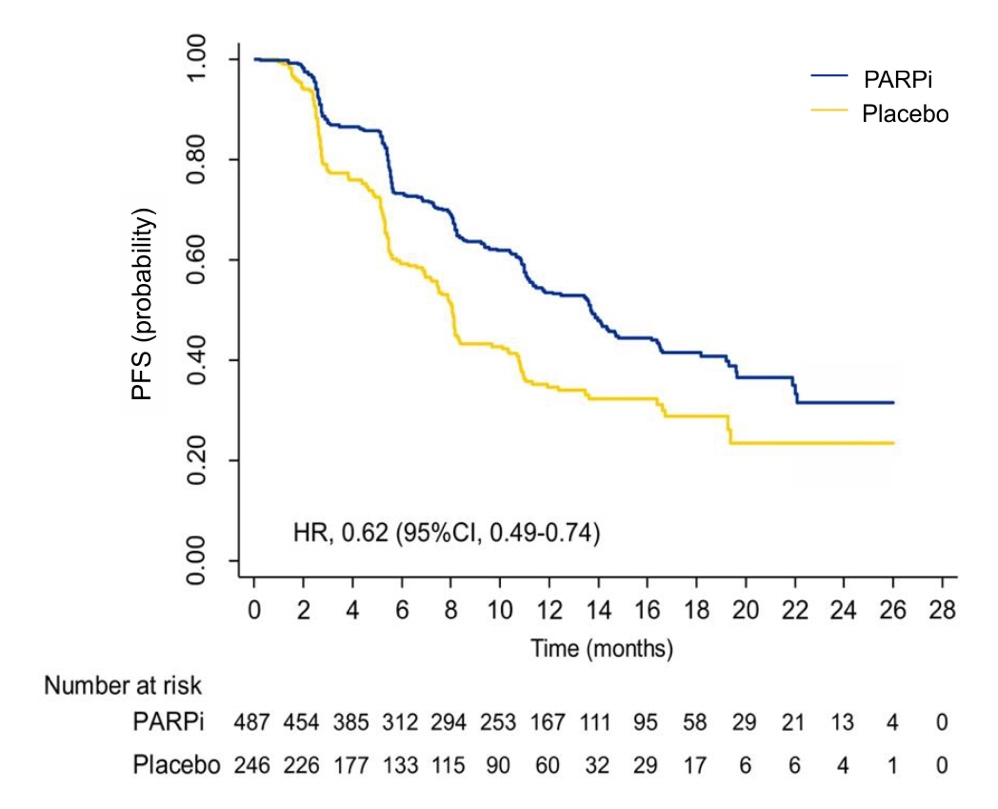


**Figure S14:** Extracted events and reconstructed progression-free survival curves of PRIMA trial (HRD positive subgroup, including BRCA mutated) by Martin AG, et al

**Figure S14-A:** Extracted events

Events in PARPi arm – 48

Events in Placebo arm – 39

**Figure S14-B:** Reconstructed progression-free survival curves


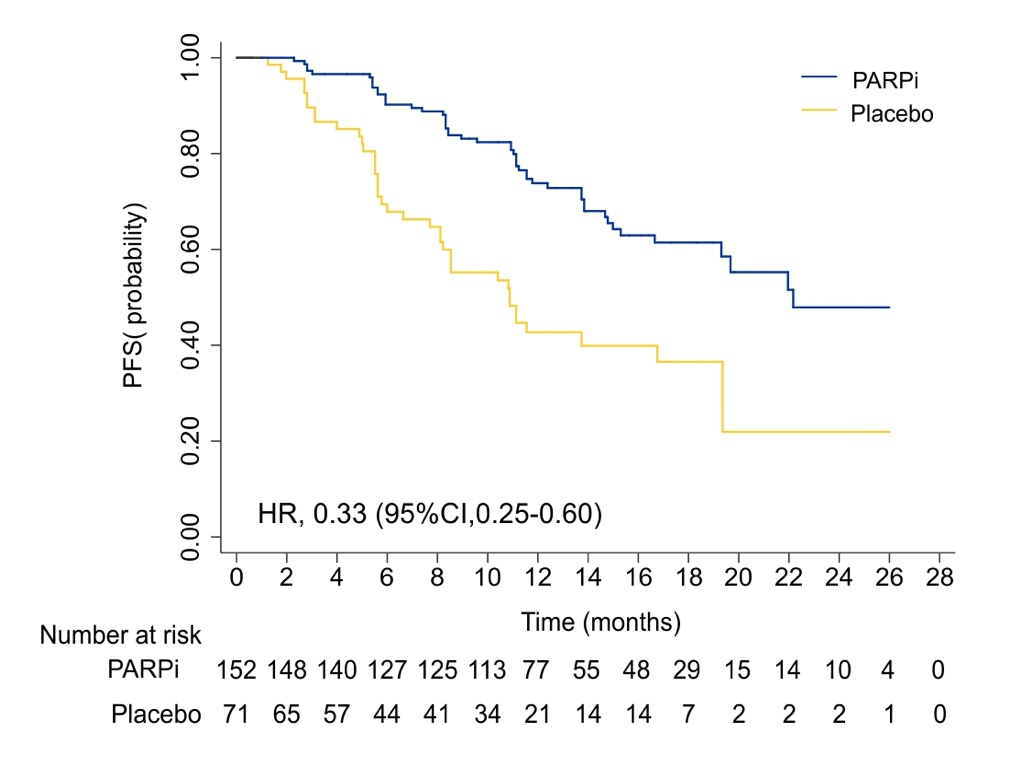


**Figure S15:** Extracted events and reconstructed progression-free survival curves of PRIMA trial (HRD positive subgroup, excluding BRCA mutated) by Martin AG, et al

**Figure S15-A:** Extracted events

Events in PARPi arm – 33

Events in Placebo arm – 32

**Figure S15-B:** Reconstructed progression-free survival curves


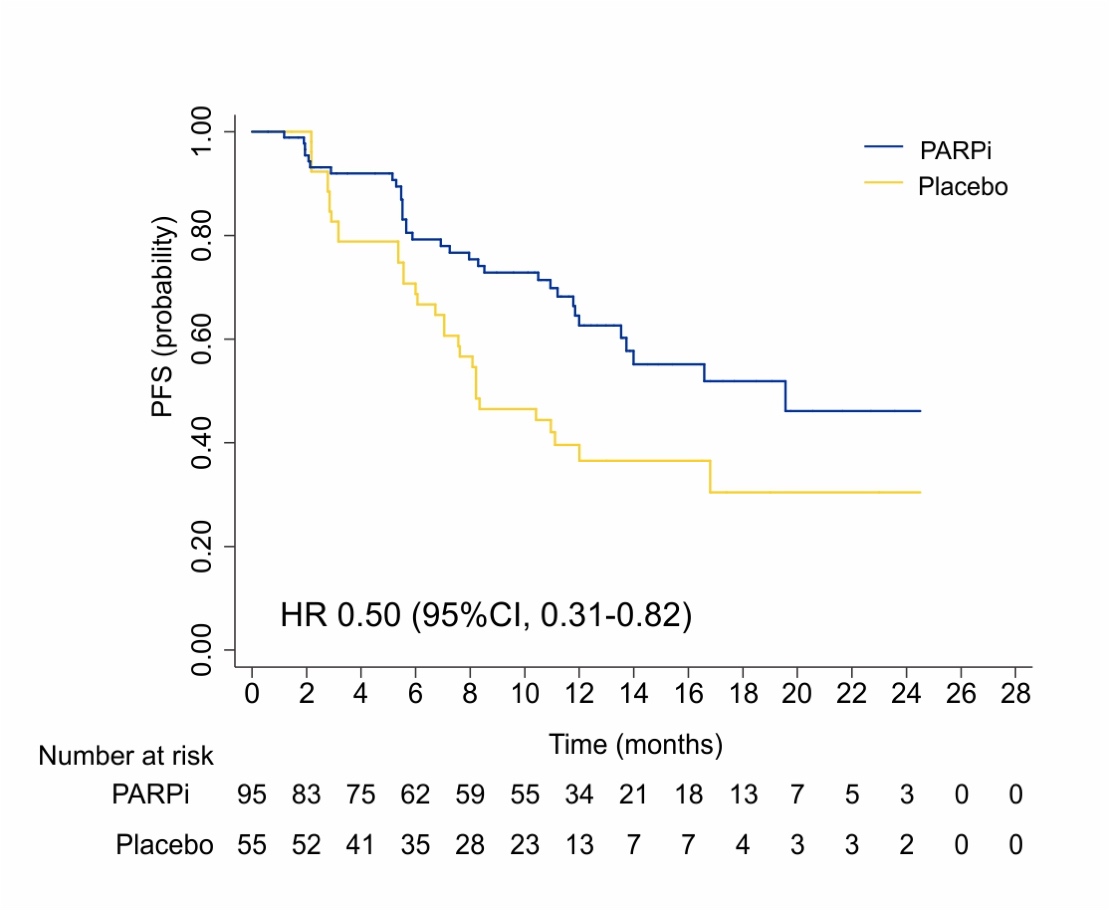


**Figure S16:** Extracted events and reconstructed progression-free survival curves of PRIMA trial (HRD negative subgroup) by Martin AG, et al

**Figure S16-A:** Extracted events

Events in PARPi arm – 109

Events in Placebo arm – 56

**Figure S16-B:** Reconstructed progression-free survival curves


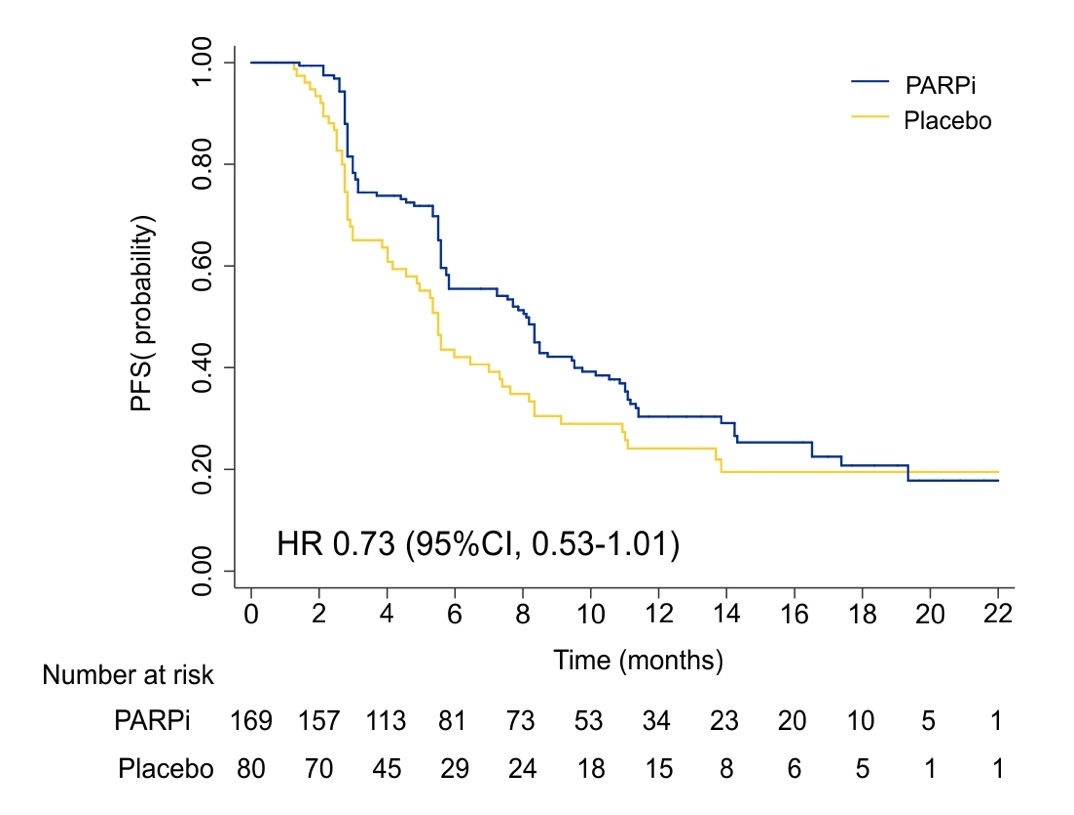


**Figure S17:** Extracted events and reconstructed progression-free survival curves of PRIMA trial (BRCA1 mutated subgroup) by Martin AG, et al

**Figure S17-A:** Extracted events

Events in PARPi arm – 39

Events in Placebo arm – 27

**Figure S17-B:** Reconstructed progression-free survival curves


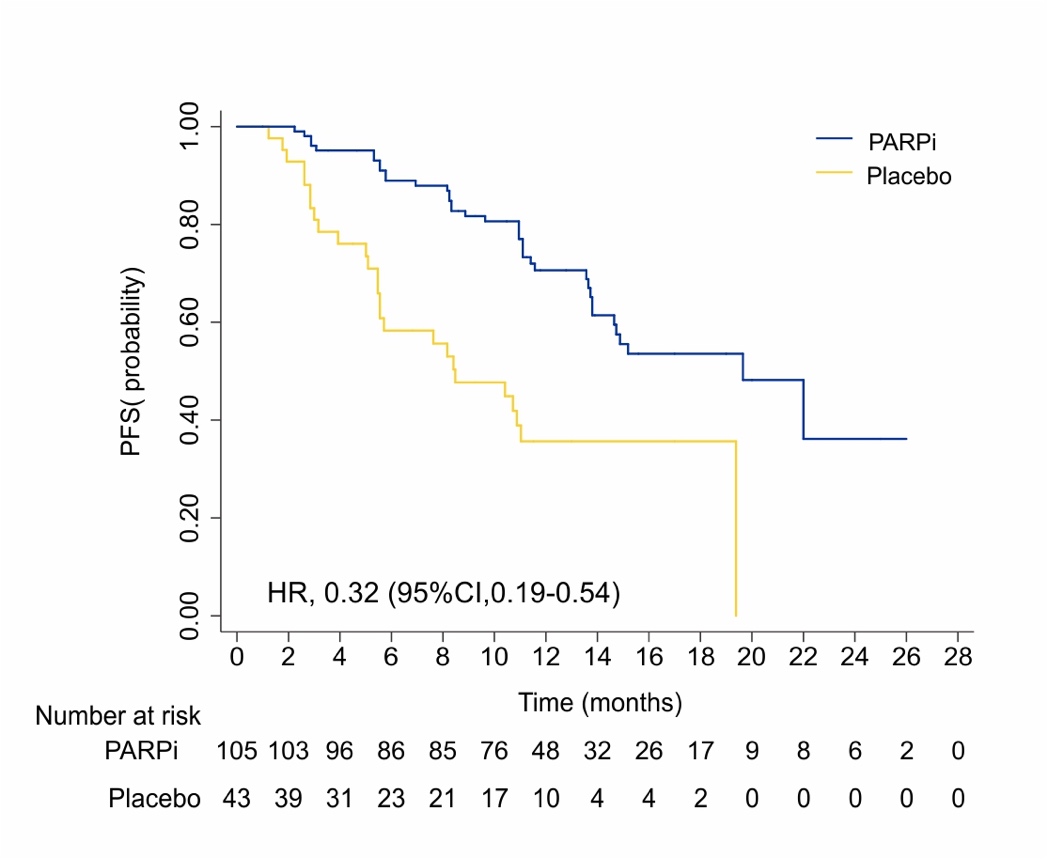


**Figure S18:** Extracted events and reconstructed progression-free survival curves of PRIMA trial (BRCA2 mutated subgroup) by Martin AG, et al

**Figure S18-A:** Extracted events

Events in PARPi arm – 9

Events in Placebo arm – 14

**Figure S18-B:** Reconstructed progression-free survival curves


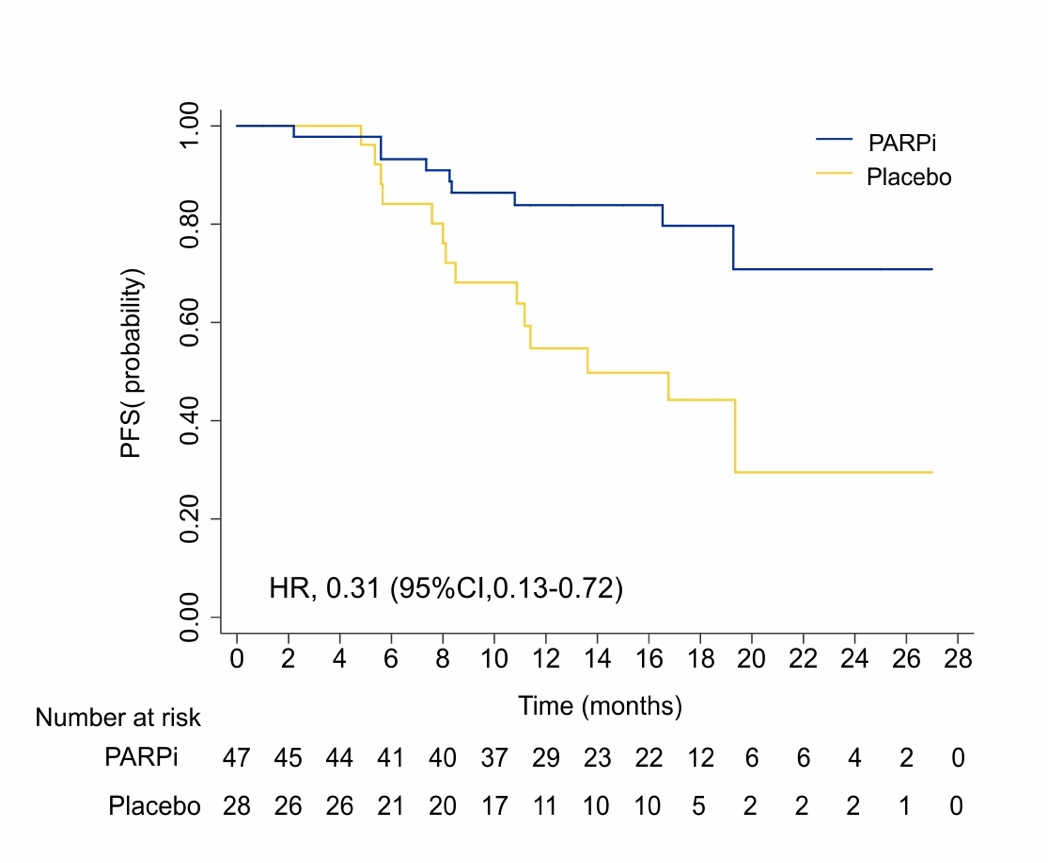


**Figure S19:** Extracted events and reconstructed progression-free survival curves of PRIMA trial (combined BRCA1 and BRCA 2 mutated subgroup) by Martin AG, et al

**Figure S19-A:** Extracted events

Events in PARPi arm – 48

Events in Placebo arm – 41

**Figure S19-B:** Reconstructed progression-free survival curves


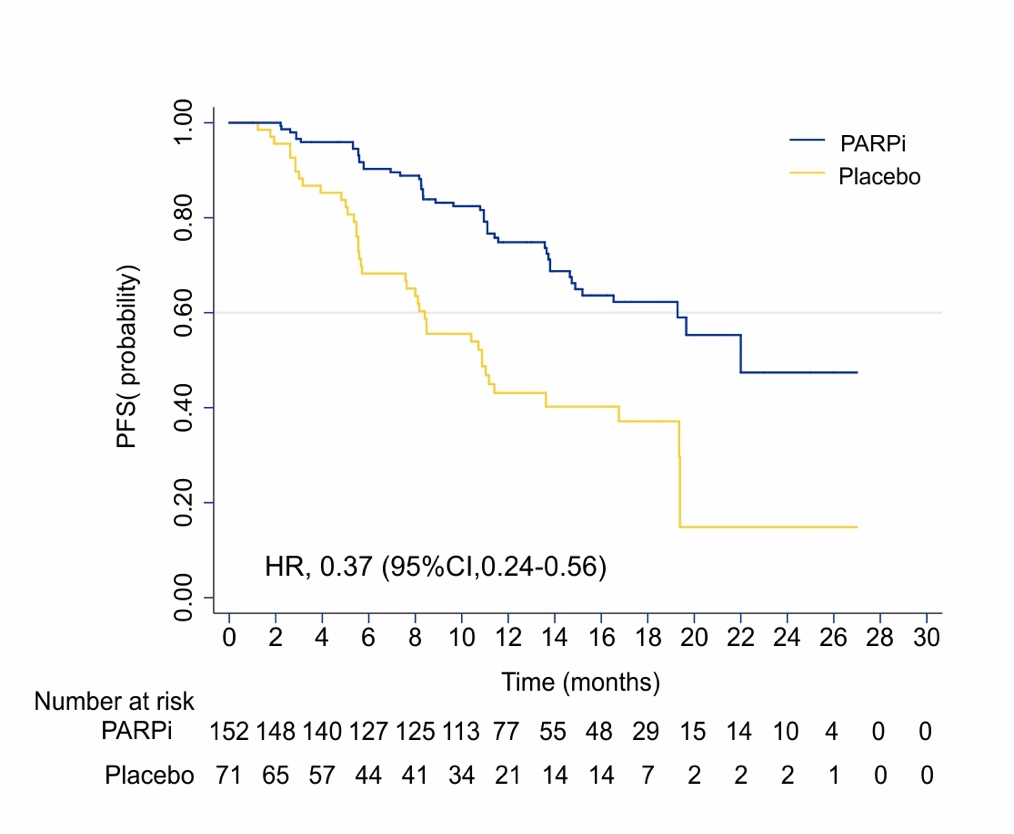


**Figure S20:** Extracted events and reconstructed progression-free survival curves of VELIA trial (whole population) by Coleman RL, et al

**Figure S20-A:** Extracted events

Events in PARPi arm – 189

Events in placebo arm – 236

**Figure S20-B:** Reconstructed progression- free survival curves


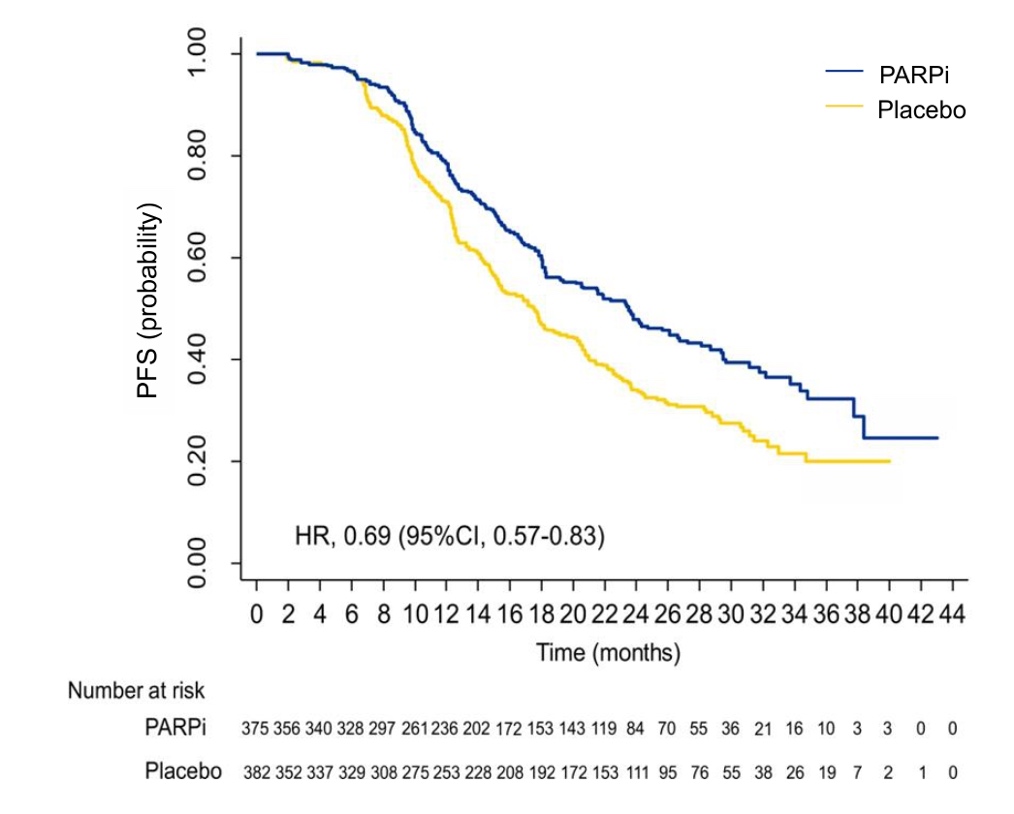


**Figure S21:** Extracted events and reconstructed progression-free survival curves of VELIA trial (HRD positive subgroup including BRCA mutated) by Coleman RL, et al

**Figure S21-A:** Extracted events

Events in PARPi arm – 87

Events in Placebo arm – 126

**Figure S21-B:** Reconstructed progression-free survival curves


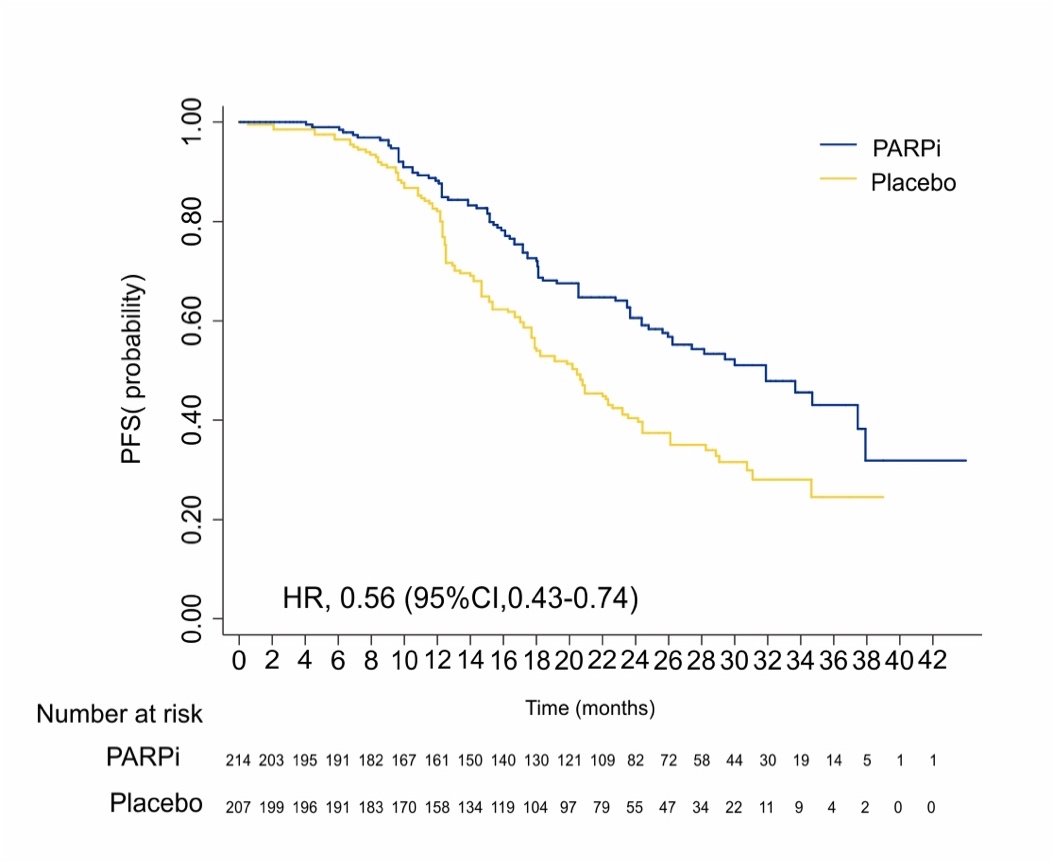


**Figure S22:** Extracted events and reconstructed progression-free survival curves of VELIA trial (HRD negative subgroup) by Coleman RL, et al

**Figure S22-A:** Extracted events

Events in PARPi arm – 80

Events in Placebo arm – 89

**Figure S22-B:** Reconstructed progression-free survival curves


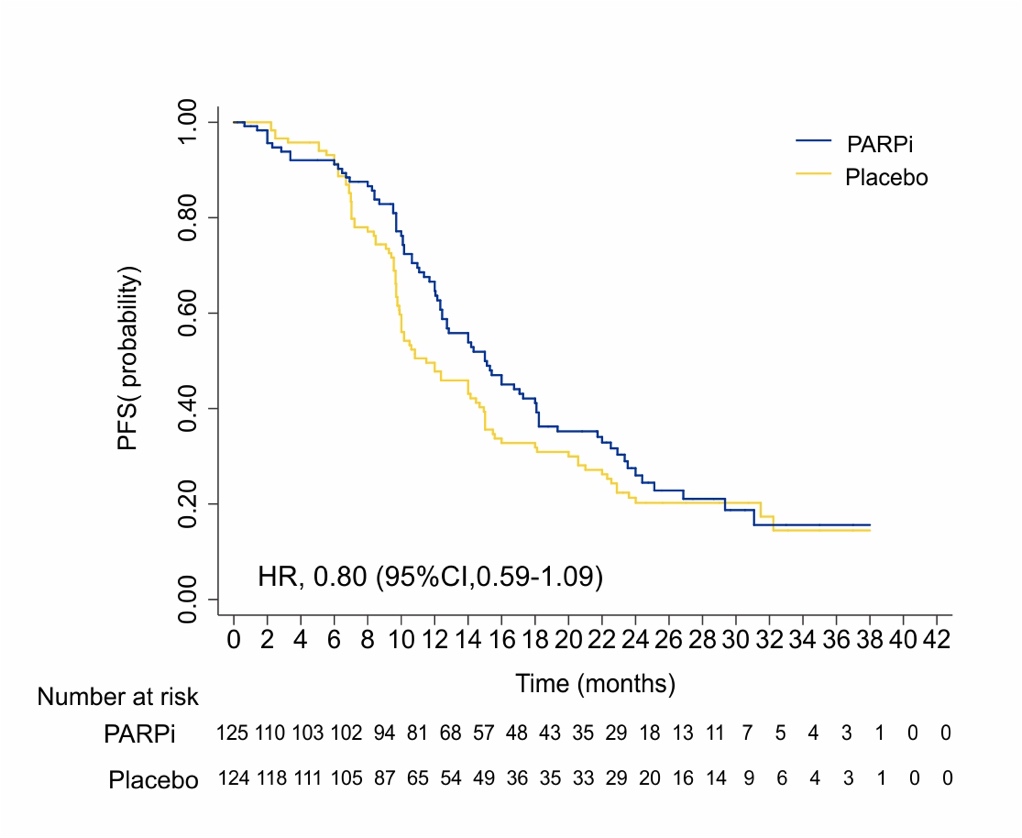


**Figure S23:** Extracted events and reconstructed progression-free survival curves of VELIA trial (BRCA1/2 mutated subgroup) by Coleman RL, et al

**Figure S23-A:** Extracted events

Events in PARPi arm – 34

Events in Placebo arm – 51

**Figure S23-B:** Reconstructed progression-free survival curves


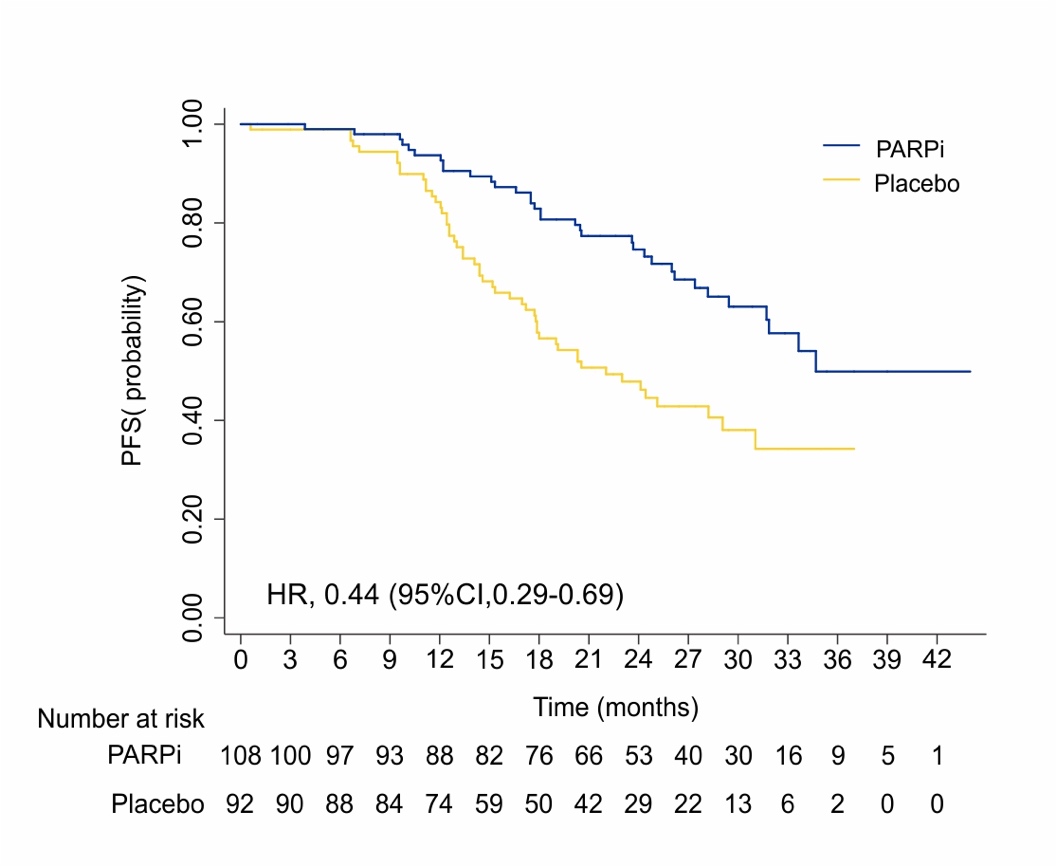


**Figure S24:** Extracted events and reconstructed progression-free survival curves of SOLO-1trial (BRCA1/2 mutated) by Moore K, et al – updated data

**Figure S24-A:** Extracted events

Events in PARPi arm – 102

Events in placebo arm – 95

**Figure S24-B:** Reconstructed progression free survival curve of SOLO-1 trial


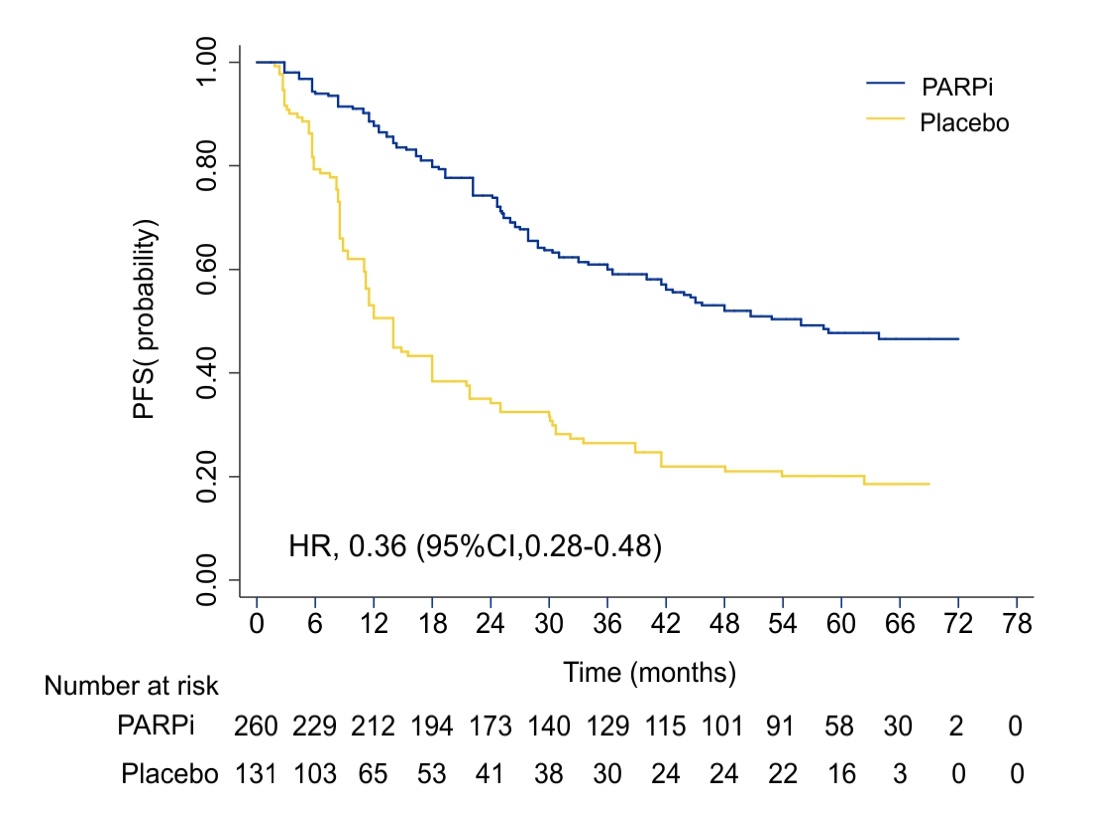

Supplement: Supplementary Material [file mmc1.docx]
